# SARS-CoV-2 Vaccination and Myocarditis in a Nordic Cohort Study of 23 Million Residents

**DOI:** 10.1001/jamacardio.2022.0583

**Published:** 2022-04-20

**Authors:** Øystein Karlstad, Petteri Hovi, Anders Husby, Tommi Härkänen, Randi Marie Selmer, Nicklas Pihlström, Jørgen Vinsløv Hansen, Hanna Nohynek, Nina Gunnes, Anders Sundström, Jan Wohlfahrt, Tuomo A. Nieminen, Maria Grünewald, Hanne Løvdal Gulseth, Anders Hviid, Rickard Ljung

**Affiliations:** 1 Department of Chronic Diseases, Norwegian Institute of Public Health, Oslo, Norway; 2 Department of Public Health and Welfare, Finnish Institute for Health and Welfare, Helsinki, Finland; 3 Department of Epidemiology Research, Statens Serum Institut, Copenhagen, Denmark; 4 Department of Epidemiology and Biostatistics, Imperial College London, London, United Kingdom; 5 Division of Licensing, Swedish Medical Products Agency, Uppsala, Sweden; 6 Health Security, Finnish Institute for Health and Welfare, Helsinki, Finland; 7 Norwegian Research Centre for Women’s Health, Oslo University Hospital, Oslo, Norway; 8 Division of Use and Information, Swedish Medical Products Agency, Uppsala, Sweden; 9 Information Services, Finnish Institute for Health and Welfare, Helsinki, Finland; 10 Department of Drug Design and Pharmacology, Pharmacovigilance Research Center, University of Copenhagen, Copenhagen, Denmark; 11 Institute of Environmental Medicine, Karolinska Institutet, Stockholm, Sweden

## Abstract

**Importance:**

Reports of myocarditis after SARS-CoV-2 messenger RNA (mRNA) vaccination have emerged.

**Objective:**

To evaluate the risks of myocarditis and pericarditis following SARS-CoV-2 vaccination by vaccine product, vaccination dose number, sex, and age.

**Design, Setting, and Participants:**

Four cohort studies were conducted according to a common protocol, and the results were combined using meta-analysis. Participants were 23 122 522 residents aged 12 years or older. They were followed up from December 27, 2020, until incident myocarditis or pericarditis, censoring, or study end (October 5, 2021). Data on SARS-CoV-2 vaccinations, hospital diagnoses of myocarditis or pericarditis, and covariates for the participants were obtained from linked nationwide health registers in Denmark, Finland, Norway, and Sweden.

**Exposures:**

The 28-day risk periods after administration date of the first and second doses of a SARS-CoV-2 vaccine, including BNT162b2, mRNA-1273, and AZD1222 or combinations thereof. A homologous schedule was defined as receiving the same vaccine type for doses 1 and 2.

**Main Outcomes and Measures:**

Incident outcome events were defined as the date of first inpatient hospital admission based on primary or secondary discharge diagnosis for myocarditis or pericarditis from December 27, 2020, onward. Secondary outcome was myocarditis or pericarditis combined from either inpatient or outpatient hospital care. Poisson regression yielded adjusted incidence rate ratios (IRRs) and excess rates with 95% CIs, comparing rates of myocarditis or pericarditis in the 28-day period following vaccination with rates among unvaccinated individuals.

**Results:**

Among 23 122 522 Nordic residents (81% vaccinated by study end; 50.2% female), 1077 incident myocarditis events and 1149 incident pericarditis events were identified. Within the 28-day period, for males and females 12 years or older combined who received a homologous schedule, the second dose was associated with higher risk of myocarditis, with adjusted IRRs of 1.75 (95% CI, 1.43-2.14) for BNT162b2 and 6.57 (95% CI, 4.64-9.28) for mRNA-1273. Among males 16 to 24 years of age, adjusted IRRs were 5.31 (95% CI, 3.68-7.68) for a second dose of BNT162b2 and 13.83 (95% CI, 8.08-23.68) for a second dose of mRNA-1273, and numbers of excess events were 5.55 (95% CI, 3.70-7.39) events per 100 000 vaccinees after the second dose of BNT162b2 and 18.39 (9.05-27.72) events per 100 000 vaccinees after the second dose of mRNA-1273. Estimates for pericarditis were similar.

**Conclusions and Relevance:**

Results of this large cohort study indicated that both first and second doses of mRNA vaccines were associated with increased risk of myocarditis and pericarditis. For individuals receiving 2 doses of the same vaccine, risk of myocarditis was highest among young males (aged 16-24 years) after the second dose. These findings are compatible with between 4 and 7 excess events in 28 days per 100 000 vaccinees after BNT162b2, and between 9 and 28 excess events per 100 000 vaccinees after mRNA-1273. This risk should be balanced against the benefits of protecting against severe COVID-19 disease.

## Key Points

**Question:**

Is SARS-CoV-2 messenger RNA (mRNA) vaccination associated with risk of myocarditis?

**Findings:**

In a cohort study of 23.1 million residents across 4 Nordic countries, risk of myocarditis after the first and second doses of SARS-CoV-2 mRNA vaccines was highest in young males aged 16 to 24 years after the second dose. For young males receiving 2 doses of the same vaccine, data were compatible with between 4 and 7 excess events in 28 days per 100 000 vaccinees after second-dose BNT162b2, and between 9 and 28 per 100 000 vaccinees after second-dose mRNA-1273.

**Meaning:**

The risk of myocarditis in this large cohort study was highest in young males after the second SARS-CoV-2 vaccine dose, and this risk should be balanced against the benefits of protecting against severe COVID-19 disease.

## Teaser

This cohort study conducted using nationwide registers assesses the risks of myocarditis and pericarditis after SARS-CoV-2 messenger RNA vaccinations in a combined population of 23.1 million individuals across Denmark, Finland, Norway, and Sweden.

## Introduction

The European Medicines Agency and European Commission have, by October 2021, approved 4 vaccines against SARS-CoV-2: BNT162b2 (Pfizer-BioNTech), mRNA-1273 (Moderna), AZD1222 (AstraZeneca), and Ad26.COV2.S (Janssen). The Nordic countries have primarily used the 2 messenger RNA (mRNA) vaccines BNT162b2 and mRNA-1273. These vaccines have been shown to be efficient and safe, although cases of myocarditis or pericarditis during the first weeks after vaccination have been reported.^1^

Case reports, surveillance data, and other reports from the US, Israel, and Canada indicate an increased risk of myocarditis after vaccination with SARS-CoV-2 mRNA vaccines, higher after the second dose, especially in younger men.^2,3,4,5,6,7,8,9^ Data from Canada and France indicate more cases of myocarditis after mRNA-1273 than after BNT162b2, but this remains to be elucidated.^10,11^

In nationwide cohort studies in Denmark, Finland, Norway, and Sweden, we evaluated the risks of myocarditis and pericarditis following SARS-CoV-2 vaccination in a combined population of 23.1 million individuals. High-quality nationwide registers enabled us to evaluate the risk by vaccine product, vaccination dose number, sex, and age.

## Methods

### Setting and Data Sources

We conducted population-based cohort studies in 4 Nordic countries (Denmark, Finland, Norway, and Sweden) using linked data from nationwide health registers on SARS-CoV-2 vaccination, myocarditis and pericarditis diagnoses, and other covariates (eMethods in the Supplement). All Nordic residents are assigned a unique personal identifier at birth or immigration, enabling deterministic linkage between registers. These countries have universal and tax-financed health care systems, and reporting to national registers is mandatory, providing near-complete follow-up of all residents over time.^12,13^ Each cohort study was analyzed separately according to a common protocol, and the results were combined by meta-analyses. On the basis of current law in each of the countries, this register-based research was conducted according to the laws, regulations, and authority permits, and informed consent from individuals was not applicable (eMethods in the Supplement).^14^ The requirement for obtaining informed consent was waived because all data are publicly available. This study followed the Strengthening the Reporting of Observational Studies in Epidemiology (STROBE) reporting guideline.

### Study Population

We included all persons who turned 12 years or older in 2021, were residents on January 1, 2017, and were alive and still residing within the country on December 27, 2020. We excluded 20 211 persons with any myocarditis or pericarditis in inpatient or outpatient hospital care from January 1, 2017, to December 26, 2020 (eMethods in the Supplement).

### SARS-CoV-2 Vaccination

**Figure 1.**
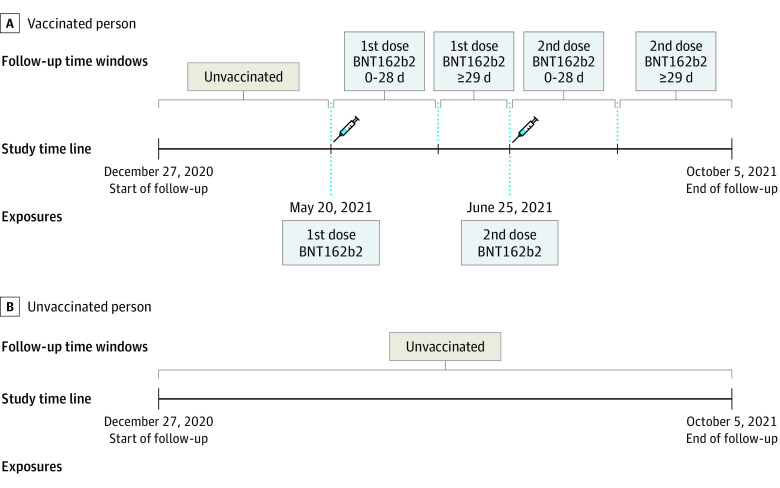
Schematic Illustrations of Follow-up Time Windows in the Cohort Study A, Example of an individual who was vaccinated with a first dose on May 20, 2021, and followed up 0 to 28 days from first dose, and vaccinated with a second dose on June 25, 2021, and followed up 0 to 28 days after a second dose. B, Example of an individual who was not vaccinated and was followed up until the end of follow-up on October 5, 2021.

The Nordic countries implemented national vaccination campaigns against SARS-CoV-2 from December 27, 2020, providing free vaccinations to all residents. Phased distribution plans were implemented, prioritizing vaccination of individuals at highest risk of COVID-19 complications (ie, nursing home residents, health care workers, and older adults). Denmark, Finland, and Norway almost exclusively used mRNA vaccines after full or partial discontinuation of AZD1222 in March 2021 because of serious but rare events of thrombosis with thrombocytopenia.^15,16^ Sweden used AZD1222 for a majority of the population older than 64 years and mRNA vaccines in other age groups. The vaccine Ad26.COV2.S had very limited use. The Nordic countries vaccinated approximately 6 times more individuals with BNT162b2 than with mRNA-1273 because of higher availability of the former vaccine. We studied risk of myocarditis and pericarditis in 28-day risk periods after the administration date of the first and second dose with BNT162b2, mRNA-1273, and AZD1222 (Figure 1). A homologous schedule was defined as receiving the same vaccine type for doses 1 and 2.

### Myocarditis and Pericarditis

We defined incident outcome events as the date of first hospital admission for myocarditis or pericarditis from December 27, 2020, onward. The primary outcome was a main or secondary diagnosis of myocarditis at discharge from inpatient hospital care. Secondary outcomes were a main or secondary diagnosis of pericarditis (inpatient hospital care) and a main or secondary diagnosis of either condition (myocarditis or pericarditis) combined from either inpatient or outpatient hospital care (eTable 1 in the Supplement).

### Covariates

We used the following covariates for adjustment and stratification: sex, age, calendar period, health care worker status, nursing home resident, and 5 comorbidities (pulmonary disease, kidney disease, autoimmune disease, cardiovascular disease or diabetes, and cancer) defined by diagnoses before the start of follow-up (eTable 2 in the Supplement). We also adjusted for verified SARS-CoV-2 infection before December 27, 2020, whereas infection after this date was a censoring event. We defined having SARS-CoV-2 as the sample date of a positive reverse transcriptase–polymerase chain reaction or lateral flow test.

### Statistical Analysis

We took advantage of the longitudinal information in our national cohorts to calculate exact unvaccinated and vaccinated person-time at risk for each individual (Figure 1). We started follow-up on December 27, 2020. Each individual was followed up until first outcome event of interest or a censoring event, defined as first occurrence of a positive test result for SARS-CoV-2 infection, receiving Ad26.COV2.S vaccine, receiving a third dose of any SARS-CoV-2 vaccine, emigration, death, or country-specific study end (latest October 5, 2021). Individuals contributed person-time as unvaccinated until the first vaccination. After each first or second dose, individuals contributed person-time in a main risk period of interest defined as day 0 up to and including day 28 (Figure 1). The resulting follow-up periods and numbers of myocarditis and pericarditis cases were aggregated for all individuals according to vaccination status (ie, unvaccinated, risk period after first dose, and risk period after second dose).

We used Poisson regression for the number of events to estimate incidence rate ratios (IRRs) with 95% CIs, comparing rates in the risk periods after vaccination with rates in unvaccinated periods. We took potential confounding factors into account by adjustment in 3 models. Model 1 included adjustment for sex and age group (12-15, 16-19, 20-24, 25-29, 30-39, 40-64, and ≥65 years). Model 2 included adjustment as in model 1 and for health care worker status, nursing home resident, and the aforementioned comorbidities. Model 3 included adjustment as in model 2 and for calendar periods (December through March, April through June, and July to the study end). We used model 2 in the main analyses, whereas models 1 and 3 were used for sensitivity analyses. We included subgroup results according to sex and age (12-15, 16-24, 25-39, and ≥40 years). Analyses were conducted in Denmark and Sweden with SAS, version 9.4 (SAS Institute Inc), in Finland with R, version 3.6.3 (R Foundation for Statistical Computing), and in Norway with Stata, version 16.0 (StataCorp LLC).

#### Meta-analyses

Meta-analyses of the IRR estimates were based on random-effects models implemented using the mixmeta package^17^ of R.^18^ We tested the homogeneity of country-specific estimates using the Cochran *Q* test,^19^ calculated the pooled incidence rates using the sum of events and person-years in the countries, and calculated the pooled excess rates using the pooled incidence rates and IRR estimates. For the CIs, we used the delta method, assuming independence of the incidence rates and IRR estimates.

#### Supplementary Analyses

In a complementary analysis, we studied incident myocarditis within 28 days following SARS-CoV-2 infection from August 1, 2020, to end of study. We also studied risk of myocarditis or pericarditis in a shorter 7-day risk period. Furthermore, among myocarditis cases, we estimated the proportion of patients discharged on day 4 or later and the proportion of cases in which the patient died within 28 days of the admission date, using the Kaplan-Meier estimator. Among myocarditis cases after vaccination, we calculated the median time from vaccination to outcome (hospital admission date).

## Results

Across 4 Nordic countries, 23 122 522 residents (49.8% male and 50.2% female) were followed up from December 27, 2020, to October 5, 2021, at the latest. By study end, 17 129 982 residents (74%) had received 2 doses and 1 681 930 residents (7%) had received 1 dose of SARS-CoV-2 vaccines. By study end, 487 751 of 1 238 004 persons (39%) aged 12 to 15 years, 2 009 995 of 2 675 558 persons (75%) aged 16 to 24 years of age, 3 736 517 of 5 046 164 persons (74%) aged 25 to 39 years, and 12 579 805 of 14 162 796 persons (89%) aged 40 years or older had received at least 1 dose of a SARS-CoV-2 vaccine (Table 1; eTable 3 in the Supplement).

**Table 1.** Number of Individuals Contributing to Unexposed and Exposed Person-Time by Vaccine Type and Vaccine Schedule^a^

Vaccine type and schedule^b^	Age group				
	≥12 y	12-15 y	16-24 y	25-39 y	≥40 y
Population at start of follow-up, No.	23 122 522	1 238 004	2 675 558	5 046 164	14 162 796
Unvaccinated by end of follow-up, No. (%)	4 308 454 (19)	750 253 (61)	665 563 (25)	1 309 647 (26)	1 582 991 (11)
At least first dose AZD1222, No. (%)	1 356 457 (6)	95 (0)	38 420 (1)	152 037 (3)	1 165 905 (8)
Only first dose AZD1222, No.	178 447	83	12 483	45 240	120 641
AZD1222/AZD1222, No.	765 655	≤5	4624	22 720	738 310
AZD1222/BNT162b2, No.	362 842	≤5	19 464	75 164	268 211
AZD1222/mRNA-1273, No.	49 513	8	1849	8913	38 743
At least first dose BNT162b2, No. (%)	15 064 585 (65)	403 915 (33)	1 674 544 (63)	2 817 934 (56)	10 168 192 (72)
Only first dose BNT162b2, No.	1 131 555	230 351	316 425	271 116	313 663
BNT162b2/BNT162b2, No.	13 315 957	172 448	1 229 590	2 294 116	9 619 803
BNT162b2/mRNA-1273, No.	615 119	1115	128 495	252 600	232 909
BNT162b2/AZD1222, No.	1954	≤5	34	102	1817
At least first dose mRNA-1273, No. (%)	2 390 870 (10)	83 741 (7)	296 865 (11)	765 518 (15)	1 244 746 (9)
Only first dose mRNA-1273, No.	371 928	61153	96 001	127 429	87 345
mRNA-1273/BNT162b2, No.	58 082	629	11 302	24 281	21 870
mRNA-1273/mRNA-1273, No.	1 960 594	21 955	189 545	613 781	1 135 313
mRNA-1273/AZD1222, No.	266	≤5	17	27	218
Other vaccinations, No. (%)	2156 (0)	0	166 (0)	1028 (0)	962 (0)

Abbreviation: mRNA, messenger RNA.

^a^ Nordic countries combined (Denmark, Finland, Norway, and Sweden).

^b^ Vaccinations included from December 27, 2020, to October 5, 2021, with males and females combined; vaccine used for the first dose given first, and vaccine used for the second dose given second.

### Myocarditis and Pericarditis During Follow-up

During the 28-day risk periods following vaccination and during unvaccinated periods (13 million person-years in total), we observed 1077 incident myocarditis cases and 1149 incident pericarditis cases. Incidence rates of myocarditis during the unvaccinated period were 9.7 per 100 000 person-years for males and 4.3 per 100 000 person-years for females (Table 2). Among individuals aged 16 to 24 years, incidence rates were 18.8 per 100 000 person-years for males and 4.4 per 100 000 person-years for females. Incidence rates of pericarditis increased with age (eTable 4 in the Supplement).

**Table 2.** Myocarditis Within 28 Days After a Dose of SARS-CoV-2 Vaccine^a^

Subgroup, exposure^b^	No. of events^c^	Follow-up, 1000 person-years	Crude incidence rate per 1000 person-years of follow-up^d^	IRR (95% CI)	No. of excess events in 28 d per 100 000 vaccinees (95% CI)
**Males, ages ≥12 y**					
Unvaccinated	520	5340.6	0.097	1 [Reference]	0 [Reference]
AZD1222	6	43.0	0.139	2.39 (1.04 to 5.48)	0.62 (0.00 to 1.24)
AZD1222/AZD1222	≤5	29.2	ND	1.29 (0.31 to 5.33)	0.12 (−0.48 to 0.72)
BNT162b2	70	560.9	0.125	1.40 (1.09 to 1.80)	0.27 (0.09 to 0.46)
BNT162b2/BNT162b2	85	495.0	0.172	2.04 (1.61 to 2.58)	0.67 (0.46 to 0.88)
BNT162b2/mRNA-1273	34	23.7	1.433	16.99 (11.51 to 25.07)	10.34 (6.86 to 13.83)
mRNA-1273	13	93.2	0.139	1.45 (0.84 to 2.52)	0.33 (−0.11 to 0.78)
mRNA-1273/mRNA-1273	53	72.3	0.733	8.55 (6.40 to 11.41)	4.97 (3.62 to 6.32)
**Males, ages 16-24 y**					
Unvaccinated	149	794.6	0.188	1 [Reference]	0 [Reference]
AZD1222	0	0.70	ND	ND	ND
AZD1222/AZD1222	0	0.10	ND	ND	ND
BNT162b2	24	63.9	0.376	2.16 (1.40 to 3.33)	1.55 (0.70 to 2.39)
BNT162b2/BNT162b2	37	41.5	0.891	5.31 (3.68 to 7.68)	5.55 (3.70 to 7.39)
BNT162b2/mRNA-1273	17	4.6	3.687	35.62 (18.87 to 67.25)	27.49 (14.41 to 40.56)
mRNA-1273	≤5	11.5	ND	2.90 (1.05 to 7.97)	1.75 (−0.20 to 3.71)
mRNA-1273/mRNA-1273	15	5.8	2.584	13.83 (8.08 to 23.68)	18.39 (9.05 to 27.72)
**Males, ages 25-39 y**					
Unvaccinated	146	1440.6	0.101	1 [Reference]	0 [Reference]
AZD1222	0	3.1	ND	ND	ND
AZD1222/AZD1222	0	0.5	ND	ND	ND
BNT162b2	17	109.2	0.156	1.62 (0.94 to 2.80)	0.46 (0.00 to 0.92)
BNT162b2/BNT162b2	15	83.9	0.179	1.75 (1.03 to 2.99)	0.59 (0.07 to 1.10)
BNT162b2/mRNA-1273	15	9.7	1.543	23.16 (12.60 to 42.59)	11.33 (5.59 to 17.07)
mRNA-1273	≤5	30.6	ND	1.27 (0.40 to 3.99)	0.16 (−0.55 to 0.86)
mRNA-1273/mRNA-1273	26	23.0	1.132	12.96 (8.23 to 20.42)	8.01 (4.92 to 11.11)
**Males, ages ≥40**					
Unvaccinated	206	2657.6	0.078	1 [Reference]	0 [Reference]
AZD1222	6	39.3	0.153	2.30 (0.99 to 5.33)	0.66 (−0.02 to 1.34)
AZD1222/AZD1222	≤5	28.6	ND	1.24 (0.30 to 5.18)	0.10 (−0.53 to 0.74)
BNT162b2	27	375.8	0.072	0.93 (0.62 to 1.40)	−0.04 (−0.28 to 0.20)
BNT162b2/BNT162b2	31	363.6	0.085	1.08 (0.74 to 1.57)	0.05 (−0.19 to 0.28)
BNT162b2/mRNA-1273	≤5	9.4	ND	3.54 (0.85 to 14.79)	1.17 (−0.58 to 2.93)
mRNA-1273	6	48	0.125	1.89 (0.84 to 4.28)	0.45 (−0.10 to 1.00)
mRNA-1273/mRNA-1273	11	43.3	0.254	3.45 (1.87 to 6.35)	1.38 (0.50 to 2.27)
**Females, ages ≥12 y**					
Unvaccinated	211	4942.2	0.043	1 [Reference]	0 [Reference]
AZD1222	≤5	64.1	ND	1.87 (0.58 to 6.03)	0.17 (−0.13 to 0.46)
AZD1222/AZD1222	≤5	31.6	ND	1.67 (0.40 to 6.97)	0.19 (−0.30 to 0.69)
BNT162b2	35	572.3	0.061	1.46 (1.01 to 2.11)	0.15 (0.02 to 0.28)
BNT162b2/BNT162b2	30	522.7	0.057	1.25 (0.77 to 2.05)	0.09 (−0.09 to 0.26)
BNT162b2/mRNA-1273	≤5	19.1	ND	9.62 (3.11 to 29.77)	1.44 (0.02 to 2.87)
mRNA-1273	≤5	90	ND	1.45 (0.35 to 5.97)	0.05 (−0.13 to 0.23)
mRNA-1273/mRNA-1273	7	71.6	0.098	2.73 (1.27 to 5.87)	0.48 (0.07 to 0.89)
**Females, ages 16-24 y**					
Unvaccinated	31	707.1	0.044	1 [Reference]	0 [Reference]
AZD1222	0	2.4	ND	ND	ND
AZD1222/AZD1222	0	0.3	ND	ND	ND
BNT162b2	≤5	63.2	ND	1.98 (0.56 to 7.01)	0.18 (−0.13 to 0.49)
BNT162b2/BNT162b2	≤5	43.9	ND	2.86 (1.10 to 7.48)	0.57 (−0.01 to 1.15)
BNT162b2/mRNA-1273	≤5	4	ND	71.70 (15.10 to 340.36)	3.74 (−1.45 to 8.93)
mRNA-1273	0	10.7	ND	ND	ND
mRNA-1273/mRNA-1273	0	6	ND	ND	ND
**Females, ages 25-39 y**					
Unvaccinated	42	1269.7	0.033	1 [Reference]	0 [Reference]
AZD1222	0	8.8	ND	ND	ND
AZD1222/AZD1222	0	1.3	ND	ND	ND
BNT162b2	≤5	105	ND	2.35 (0.90 to 6.12)	0.21 (−0.03 to 0.45)
BNT162b2/BNT162b2	≤5	85	ND	2.35 (0.89 to 6.25)	0.26 (−0.04 to 0.55)
BNT162b2/mRNA-1273	0	7.5	ND	ND	ND
mRNA-1273	0	27.7	ND	ND	ND
mRNA-1273/mRNA-1273	≤5	21	ND	7.31 (2.16 to 24.78)	0.95 (−0.14 to 2.03)
**Females, ages ≥40 y**					
Unvaccinated	137	2541.6	0.054	1 [Reference]	0 [Reference]
AZD1222	≤5	52.9	ND	ND	ND
AZD1222/AZD1222	≤5	30	ND	ND	ND
BNT162b2	27	392.5	0.069	1.37 (0.90 to 2.08)	0.14 (−0.03 to 0.31)
BNT162b2/BNT162b2	20	388.1	0.052	1.02 (0.63 to 1.65)	0.01 (−0.18 to 0.20)
BNT162b2/mRNA-1273	≤5	7.5	ND	8.12 (1.83 to 36.00)	1.79 (−0.72 to 4.29)
mRNA-1273	≤5	48.5	ND	4.68 (0.60 to 36.45)	0.12 (−0.13 to 0.38)
mRNA-1273/mRNA-1273	≤5	44.4	ND	3.03 (1.10 to 8.31)	0.46 (−0.05 to 0.97)

Abbreviations: IRR, adjusted incidence rate ratio; mRNA, messenger RNA; ND, not determined.

^a^ The IRRs and excess events in 28 days per 100 000 vaccinees, according to sex and age. The IRRs for model 2, adjusted for age group, sex, previous SARS-CoV-2 infection, health care worker status, nursing home resident, and comorbidity variables; for other models see eFigure 2 and eTable 5 in the Supplement.

^b^ Vaccine doses listed in sequential order.

^c^ On rows without cases, only follow-up data are shown.

^d^ On rows with 5 or fewer cases, incident rate is not given.

### Vaccination and Myocarditis

During the 28-day risk period, we observed 105 myocarditis cases following administration of the first dose of BNT162b2 and 115 myocarditis cases following the second dose. We also observed 15 myocarditis cases following administration of the first dose of mRNA-1273 and 60 myocarditis cases following the second dose.

Adjusted IRRs comparing the 28-day risk periods following first and second doses compared with unvaccinated periods were 1.38 (95% CI, 1.12-1.69) for the first dose of BNT162b2 and 1.75 (95% CI, 1.43-2.14) for the second dose, and 1.16 (95% CI, 0.69-1.93) for the first dose of mRNA-1273 and 6.57 (95% CI, 4.64-9.28) for the second dose. Among males, after the first and second doses, adjusted IRRs were 1.40 (95% CI, 1.09-1.80) for the first dose of BNT162b2 and 2.04 (95% CI, 1.61-2.58) for the second dose, and 1.45 (95% CI, 0.84-2.52) for the first dose of mRNA-1273 and 8.55 (95% CI, 6.40-11.41) for the second dose. Among females, following the first and second doses, adjusted IRRs were 1.46 (95% CI, 1.01-2.11) for the first dose of BNT162b2 and 1.25 (95% CI, 0.77-2.05) for the second dose, and 1.45 (95% CI, 0.35-5.97) for the first dose of mRNA-1273 and 2.73 (95% CI, 1.27-5.87) for the second dose.

**Figure 2.**
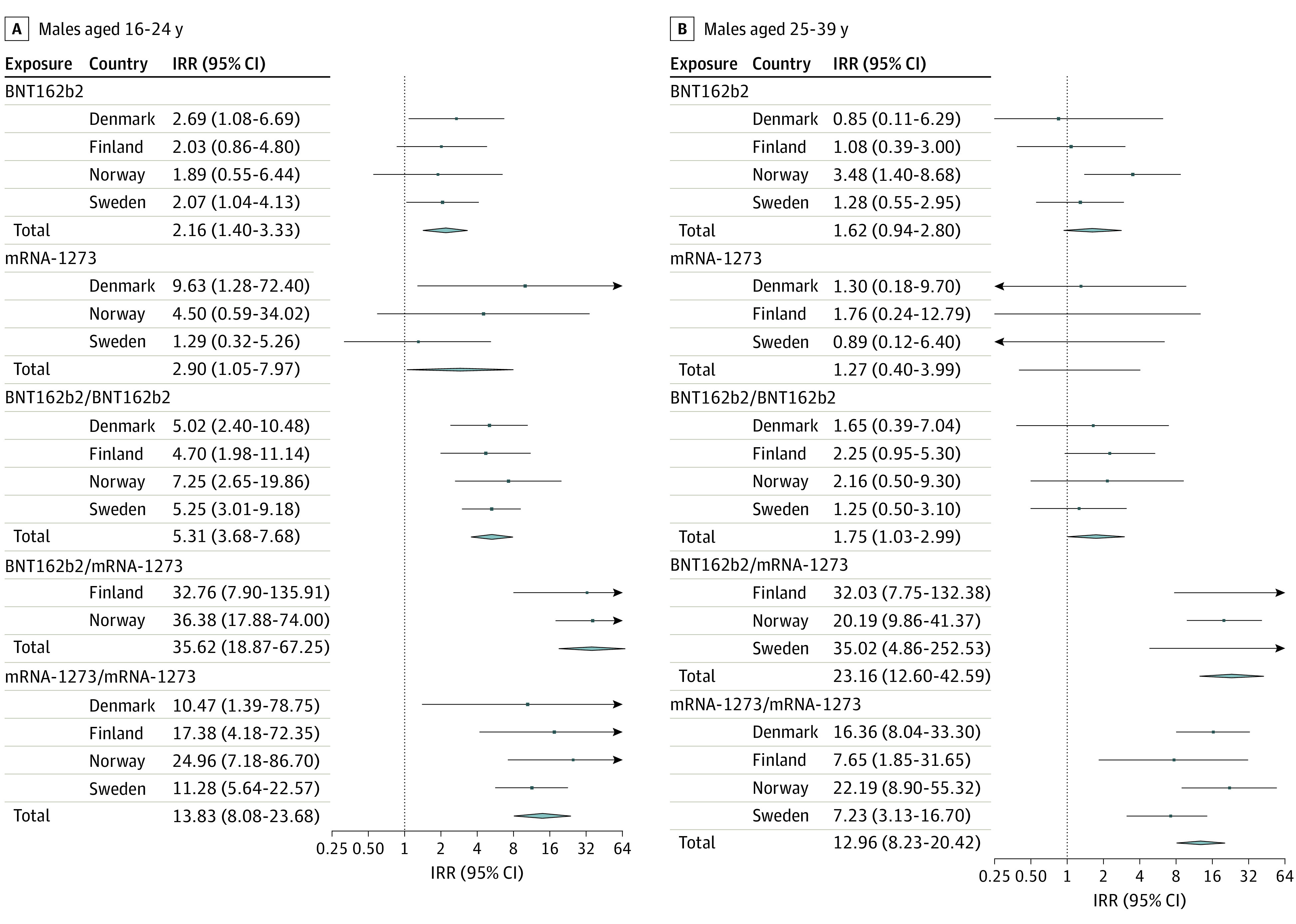
Myocarditis Within 28 Days After SARS-CoV-2 Vaccination in 4 Nordic Countries Among Males Aged 16 to 39 Years, With Pooled Estimates Squares represent incidence rate ratios (IRRs) with 95% CIs; square size, country weight; and diamonds, pooled estimates with 95% CIs. A single vaccine name indicates first dose of that vaccine (eg, BNT162b2) and the risk of the outcome after the first dose. Vaccine names in combination indicate a vaccine schedule of first dose of the first vaccine and a second dose of the second vaccine (eg, BNT162b2, BNT162b2) and the risk of the outcome after the second dose. Model 2 adjusted for age group and sex, previous SARS-CoV-2 infection, health care worker status, nursing home resident, and comorbidity variables.

**Figure 3.**
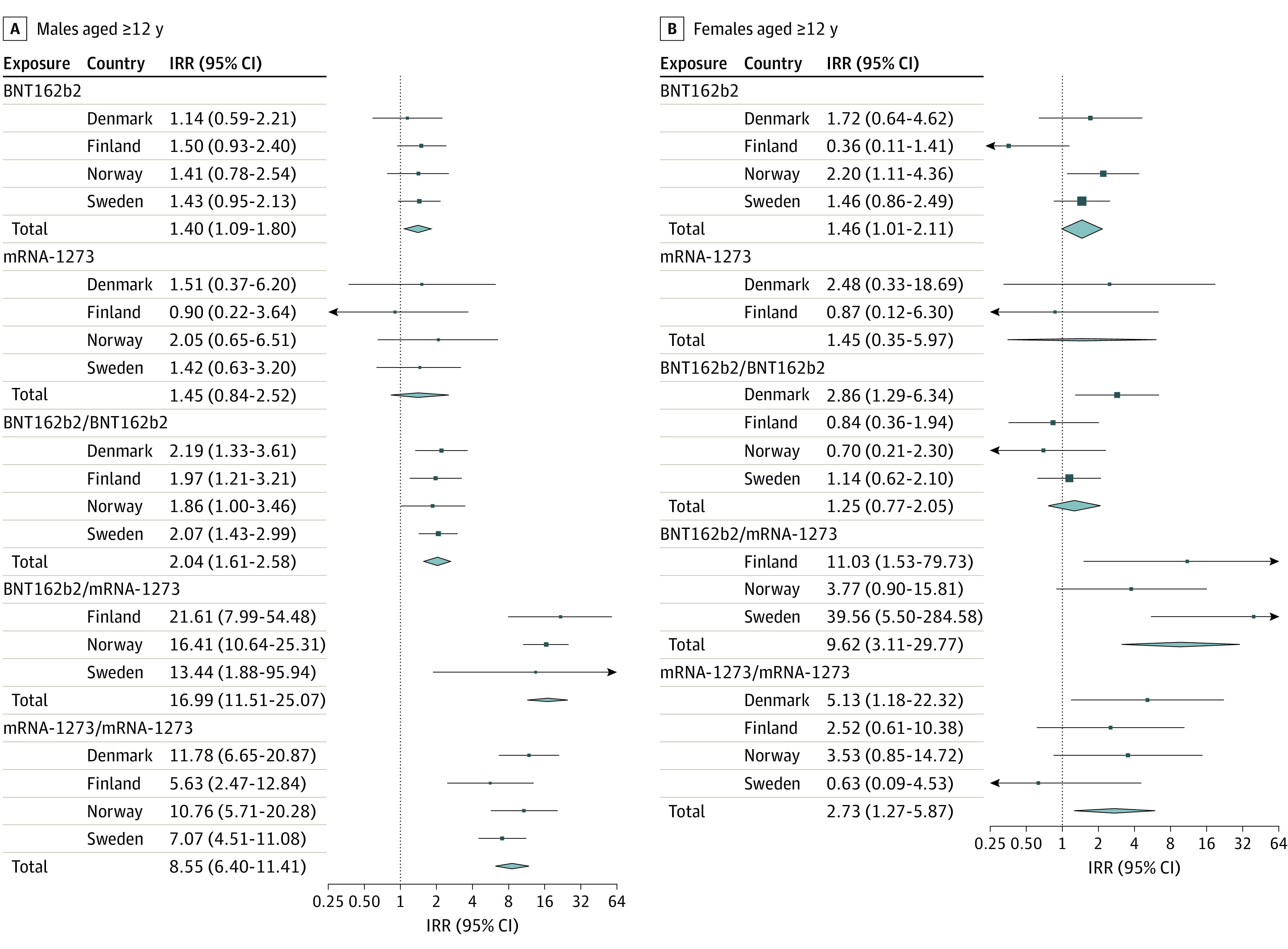
Myocarditis Within 28 Days After SARS-CoV-2 Vaccination in 4 Nordic Countries Among Males and Females Aged 12 Years or Older, With Pooled Estimates Squares represent incidence rate ratios (IRRs) with 95% CIs; square size, country weight; and diamonds, pooled estimates with 95% CIs. A single vaccine name indicates first dose of that vaccine (eg, BNT162b2) and the risk of the outcome after the first dose. Vaccine names in combination indicate a vaccine schedule of first dose of the first vaccine and a second dose of the second vaccine (eg, BNT162b2, BNT162b2) and the risk of the outcome after the second dose. Model 2 adjusted for age group and sex, previous SARS-CoV-2 infection, health care worker status, nursing home resident, and comorbidity variables.

Among males 16 to 24 years of age, the adjusted IRRs for myocarditis were 5.31 (95% CI, 3.68-7.68) for a second dose of BNT162b2 and 13.83 (95% CI, 8.08-23.68) for a second dose of mRNA-1273. For females, the comparative adjusted IRRs were lower (Table 2, Figure 2, Figure 3; eFigure 1 in the Supplement).

We also estimated the excess numbers of myocarditis events per 100 000 vaccinees in the 28-day risk periods. Among all males, these numbers were 0.27 (95% CI, 0.09-0.46) events after the first dose of BNT162b2 and 0.67 (95% CI, 0.46-0.88) events after the second dose, and 0.33 (95% CI, −0.11 to 0.78) events after the first dose of mRNA-1273 and 4.97 (95% CI, 3.62-6.32) events after the second dose. Among all females, the excess numbers of events per 100 000 vaccinees in the 28-day risk periods were 0.15 (95% CI, 0.02-0.28) events after the first dose of BNT162b2 and 0.09 (95% CI, −0.09 to 0.26) events after the second dose, and 0.05 (95% CI, −0.13 to 0.23) events after the first dose of mRNA-1273 and 0.48 (95% CI, 0.07-0.89) events after the second dose (Table 2).

Among males 16 to 24 years of age, the excess number of myocarditis events per 100 000 vaccinees in the 28-day risk periods after the first dose of BNT162b2 was 1.55 (95% CI, 0.70-2.39) events and after the second dose was 5.55 (95% CI, 3.70-7.39) events, and it was 1.75 (95% CI, −0.20 to 3.71) events after the first dose of mRNA-1273 and 18.39 (95% CI, 9.05-27.72) events after the second dose (Table 2).

For a heterologous schedule (1 dose with BNT162b2 and the other dose with mRNA-1273), 38 myocarditis cases (34 males) occurred following the second dose, with an excess number of events in males of 10.34 (95% CI, 6.86-13.83) events. In males aged 16 to 24 years, 17 myocarditis cases occurred, with an excess number of events of 27.49 (95% CI, 14.41-40.56) events (Table 2).

### Vaccination and Pericarditis

Pericarditis in males followed a pattern similar to myocarditis by vaccine product and age but with lower IRRs. Pericarditis was rare in females aged 12 to 39 years. Among males aged 16 to 24 years of age, the excess number of pericarditis events within the 28-day risk period was 7.39 per 100 000 vaccinees (95% CI, 1.46-13.32) events for the second dose of mRNA-1273 (eTables 4 and 5 in the Supplement).

### Vaccination and Myocarditis or Pericarditis Combined

The IRRs of myocarditis or pericarditis combined among males aged 16 to 24 years were slightly higher than those of myocarditis (Table 3). In males aged 25 to 39 years, the IRRs were generally lower. Among females aged 16 to 24 years, the IRRs were similar to those for males but with fewer events. Among males aged 12 to 15 years, the crude IRR was based on very few events among the vaccinated population (eTable 6 in the Supplement).

**Table 3.** Myocarditis or Pericarditis Combined Within 28 Days After a Dose of SARS-CoV-2 Vaccine, According to Sex and Age^a^

Subgroup, exposure^b^	No. of events^c^	Follow-up, 1000 person-years	Crude incidence rate per 1000 person-years of follow-up^d^	IRR (95% CI)	No. of excess events in 28 d per 100 000 vaccinees (95% CI)
**Males, aged ≥12 y**					
Unvaccinated	1394	5340.4	0.261	1 [Reference]	0 [Reference]
AZD1222	18	43	0.418	1.47 (0.91 to 2.36)	1.02 (−0.12 to 2.16)
AZD1222/AZD1222	10	29.2	0.342	1.22 (0.64 to 2.30)	0.47 (−0.93 to 1.87)
BNT162b2	213	560.8	0.380	1.38 (1.19 to 1.60)	0.80 (0.48 to 1.13)
BNT162b2/BNT162b2	227	495	0.459	1.65 (1.43 to 1.91)	1.39 (1.04 to 1.74)
BNT162b2/mRNA-1273	57	23.7	2.402	8.21 (6.20 to 10.88)	16.18 (11.94 to 20.43)
mRNA-1273	30	93.2	0.322	1.17 (0.82 to 1.68)	0.36 (−0.41 to 1.14)
mRNA-1273/mRNA-1273	93	72.3	1.287	4.63 (3.75 to 5.72)	7.74 (6.10 to 9.37)
**Males, aged 16-24 y**					
Unvaccinated	271	794.5	0.341	1 [Reference]	0 [Reference]
AZD1222	≤5	0.7	ND	8.69 (0.98 to 77.17)	10.34 (−10.14 to 30.81)
AZD1222/AZD1222	0	0.1	ND	ND	ND
BNT162b2	41	63.9	0.642	1.94 (1.39 to 2.70)	2.38 (1.27 to 3.49)
BNT162b2/BNT162b2	59	41.5	1.420	4.20 (3.15 to 5.58)	8.30 (6.05 to 10.54)
BNT162b2/mRNA-1273	24	4.6	5.206	20.04 (12.29 to 32.69)	37.94 (22.73 to 53.14)
mRNA-1273	7	11.5	0.611	2.20 (1.03 to 4.67)	2.55 (0.07 to 5.03)
mRNA-1273/mRNA-1273	22	5.8	3.790	11.36 (7.32 to 17.65)	26.51 (15.38 to 37.64)
**Males, aged 25-39 y**					
Unvaccinated	344	1440.5	0.239	1 [Reference]	0 [Reference]
AZD1222	≤5	3.1	ND	2.62 (0.35 to 19.35)	1.54 (−2.03 to 5.10)
AZD1222/AZD1222	0	0.5	ND	ND	ND
BNT162b2	43	109.2	0.394	1.62 (1.02 to 2.56)	1.15 (0.23 to 2.08)
BNT162b2/BNT162b2	41	83.9	0.489	2.10 (1.49 to 2.97)	1.96 (1.10 to 2.83)
BNT162b2/mRNA-1273	27	9.7	2.778	11.47 (7.50 to 17.55)	19.45 (12.07 to 26.83)
mRNA-1273	12	30.6	0.392	1.64 (0.92 to 2.93)	1.17 (−0.08 to 2.43)
mRNA-1273/mRNA-1273	42	23	1.829	7.33 (5.27 to 10.19)	12.11 (8.40 to 15.83)
**Males, aged ≥40 y**					
Unvaccinated	748	2657.5	0.282	1 [Reference]	0 [Reference]
AZD1222	16	39.3	0.407	1.30 (0.78 to 2.15)	0.72 (−0.55 to 1.98)
AZD1222/AZD1222	10	28.6	0.350	1.13 (0.59 to 2.13)	0.30 (−1.23 to 1.83)
BNT162b2	125	375.7	0.333	1.10 (0.91 to 1.33)	0.23 (−0.22 to 0.67)
BNT162b2/BNT162b2	122	363.6	0.336	1.09 (0.90 to 1.32)	0.22 (−0.24 to 0.67)
BNT162b2/mRNA-1273	6	9.4	0.640	2.30 (1.02 to 5.20)	2.77 (−0.05 to 5.59)
mRNA-1273	10	48	0.208	0.74 (0.40 to 1.38)	−0.56 (−1.95 to 0.83)
mRNA-1273/mRNA-1273	28	43.3	0.647	2.25 (1.54 to 3.29)	2.76 (1.44 to 4.08)
**Females, aged ≥12 y**					
Unvaccinated	619	4942.1	0.125	1 [Reference]	0 [Reference]
AZD1222	10	64.1	0.156	1.24 (0.66 to 2.35)	0.23 (−0.40 to 0.87)
AZD1222/AZD1222	≤5	31.6	ND	0.74 (0.27 to 2.00)	−0.34 (−1.68 to 1.01)
BNT162b2	96	572.3	0.168	1.15 (0.93 to 1.43)	0.17 (−0.07 to 0.42)
BNT162b2/BNT162b2	102	522.7	0.195	1.26 (0.95 to 1.68)	0.31 (−0.03 to 0.66)
BNT162b2/mRNA-1273	15	19.1	0.787	6.64 (3.90 to 11.30)	5.13 (2.49 to 7.77)
mRNA-1273	22	90	0.244	1.96 (1.28 to 3.00)	0.92 (0.36 to 1.48)
mRNA-1273/mRNA-1273	28	71.6	0.391	2.88 (1.87 to 4.45)	1.96 (1.10 to 2.81)
**Females, aged 16-24 y**					
Unvaccinated	6	333	0.018	1 [Reference]	0 [Reference]
AZD1222	0	0	ND	ND	ND
AZD1222/AZD1222	0	0	ND	ND	ND
BNT162b2	0	8.5	ND	ND	ND
BNT162b2/BNT162b2	0	5.6	ND	ND	ND
BNT162b2/mRNA-1273	0	0	ND	ND	ND
mRNA-1273	≤5	3.2	ND	13.12 (1.19 to 144.65)	2.24 (−2.17 to 6.65)
mRNA-1273/mRNA-1273	0	0.2	ND	ND	ND
**Females, aged 25-39 y**					
Unvaccinated	66	707.1	0.093	1 [Reference]	0 [Reference]
AZD1222	0	2.4	ND	ND	ND
AZD1222/AZD1222	0	0.3	ND	ND	ND
BNT162b2	7	63.2	0.111	1.20 (0.55 to 2.63)	0.14 (−0.42 to 0.71)
BNT162b2/BNT162b2	10	43.9	0.228	2.49 (1.27 to 4.88)	1.05 (0.24 to 1.85)
BNT162b2/mRNA-1273	≤5	4	ND	21.19 (7.85 to 57.19)	9.05 (1.10 to 16.99)
mRNA-1273	≤5	10.7	ND	6.34 (2.26 to 17.77)	2.41 (0.00 to 4.82)
mRNA-1273/mRNA-1273	6	6	1.001	24.26 (10.03 to 58.68)	7.36 (1.46 to 13.26)
**Females, aged ≥40 y**					
Unvaccinated	93	1269.7	0.073	1 [Reference]	0 [Reference]
AZD1222	0	8.8	ND	ND	ND
AZD1222/AZD1222	0	1.3	ND	ND	ND
BNT162b2	12	105	0.114	2.09 (1.13 to 3.88)	0.46 (0.09 to 0.82)
BNT162b2/BNT162b2	16	85	0.188	2.84 (1.11 to 7.25)	0.94 (0.27 to 1.60)
BNT162b2/mRNA-1273	≤5	7.5	ND	12.33 (4.51 to 33.67)	4.71 (0.56 to 8.87)
mRNA-1273	6	27.7	0.217	6.50 (2.71 to 15.56)	1.41 (0.26 to 2.56)
mRNA-1273/mRNA-1273	8	21	0.381	6.48 (3.12 to 13.45)	2.47 (0.73 to 4.21)

Abbreviations: IRR, adjusted incidence rate ratio; mRNA, messenger RNA; ND, not determined.

^a^ The IRRs and excess events in 28 days per 100 000 vaccinees, according to sex and age. The IRRs for model 2 adjusted for age group, sex, previous SARS-CoV-2 infection, health care worker status, nursing home resident, and comorbidity variables; for other models see eTable 5 in the Supplement.

^b^ Vaccine doses listed in sequential order.

^c^ On rows without cases, only follow-up data are shown.

^d^ On rows with 5 or fewer cases, incidence rate is not given.

### SARS-CoV-2 Infection and Myocarditis

During the 28-day risk period after a positive SARS-CoV-2 test, there were 73 myocarditis cases. Excess events of myocarditis were 3.26 (95% CI, 1.90-4.61) events per 100 000 individuals with a positive test result among all males, and 1.37 (95% CI, −0.14 to 2.87) events per 100 000 individuals with a positive test result among males aged 16 to 24 years (eTable 7 in the Supplement).

### Supplementary Analyses

The IRRs and excess rates were slightly attenuated when model 1 was complemented by other covariates (model 2) and further attenuated when calendar period was added (model 3) (eFigure 2 and eTable 5 in the Supplement). Among males aged 16 to 24 years, adjustment for calendar period (model 3) yielded unstable point estimates with wide CIs for the second dose of mRNA-1273. Heterogeneity of the analyses across countries was not statistically significant (eFigure 2 in the Supplement); thus, we present the results as pooled 4-country estimates of IRRs and excess rates.

Of the 213 myocarditis cases in the 28-day risk window after a second dose of SARS-CoV-2 mRNA vaccination, 135 events occurred within the first week, yielding higher IRRs in the 7-day risk period (Table 2; eTable 8 in the Supplement). Among males aged 16 to 24 years, the adjusted IRRs were 12.50 (8.24-18.96) for a second dose of BNT162b2 and 38.29 (21.95-66.80) for a second dose of mRNA-1273.

For males aged 12 to 39 years, country-specific median time to hospital admission for myocarditis cases was 3 to 7 days (eTable 9 in the Supplement). Comorbid conditions did not differ markedly between vaccinated and unvaccinated myocarditis cases (eTable 10 in the Supplement). Median hospital length of stay was 4 to 5 days for both vaccinated and unvaccinated cases (eTable 11 in the Supplement). For all age groups, the 28-day mortality of the unvaccinated cases with myocarditis was 0.8% (95% CI, 0.3%-2.0%) and ranged from 0.2% (95% CI, 0.0%-0.4%) after the second dose of BNT162b2 to 4.5% (95% CI, 0.0%-13.2%) after the second dose of mRNA-1273; there were no deaths among cases for patients younger than 40 years (eTable 11 in the Supplement).

## Discussion

This cohort study of 23.1 million residents across 4 Nordic countries showed higher rates of myocarditis and pericarditis within 28 days after being vaccinated with SARS-CoV-2 mRNA vaccines compared with being unvaccinated. The risks of myocarditis and pericarditis were highest within the first 7 days of being vaccinated, were increased for all combinations of mRNA vaccines, and were more pronounced after the second dose. A second dose of mRNA-1273 had the highest risk of myocarditis and pericarditis, with young males aged 16 to 24 years having the highest risk.

Myocarditis after mRNA vaccination was rare in this study cohort and even among young males. The risk of myocarditis following the mRNA vaccines has been evaluated by the US Food and Drug Administration, which concluded that the benefits of vaccination outweigh the risks and fully authorized the use of mRNA-1273 in persons 18 years or older and BNT162b2 in persons 16 years or older. In addition, BNT162b2 is authorized for emergency use in children 5 years or older.^20,21^ The European Medicines Agency concluded that the benefits of vaccination outweigh the risks and approved mRNA-1273 for use in persons 12 years or older and BNT162b2 for those 5 years or older.^22,23^ In addition, a comment published by the American College of Cardiology^24^ evaluated vaccine-associated myocarditis risk and concluded that the benefits of vaccination outweigh the risks. As of January 2022, there have been nearly 5.8 million deaths associated with COVID-19 worldwide since the start of the pandemic.^25^ All currently available SARS-CoV-2 mRNA vaccines are highly effective against severe COVID-19 and provide some protection against transmission and infection.^26,27,28^ There is some evidence that the mRNA-1273 vaccine, possibly owing to its higher concentration of mRNA, is associated with increased immunogenicity and effectiveness.^29,30^ This more profound immune response could be one reason for the higher risk of myocarditis, but this hypothesis needs to be investigated further.

Our findings are consistent with higher risk after the second dose and higher risk in young males.^2,3,10,11,31,32,33,34,35,36^ Excess events within 28 days in males aged 16 to 24 years of 5.55 events per 100 000 vaccinees after the second dose with BNT162b2 and 18.39 events per 100 000 vaccinees after the second dose with mRNA-1273 are among the highest reported.^3,4,32,33^ Our finding of a higher risk of myocarditis after mRNA-1273 than after BNT162b2 in this group is in line with data from the US, Canada, France, and England.^5,10,11,33,35^ In comparison with previous studies, we had the advantage of data analyzed according to a common protocol from 4 different countries, and that showed similar directions of associations, despite considerable differences in prior SARS-CoV-2 infection levels and lockdown policies.

### Strengths and Limitations

The main strengths of our study include the population-based cohort design in 4 Nordic countries, large sample size, near-complete follow-up, and independent ascertainment of vaccinations and diagnoses from nationwide registers with mandatory reporting. The findings in the meta-analyses were supported by consistent findings across all 4 countries, despite some country-specific differences in data sources, SARS-CoV-2 transmission, testing activities, and vaccination schedules.

There are also some limitations of the study. We defined events as an inpatient hospital admission with a corresponding main or secondary discharge diagnosis of myocarditis or pericarditis. Diagnostic codes have been shown to have 85% positive predictive value among patients younger than 60 years.^37^ Thus, without access to data on clinical measures, such as troponin levels, diagnostic imaging results, and endomyocardial biopsy, we studied myocarditis as diagnosed in clinical practice and could therefore not assess how many of these patients fulfilled all criteria for receiving a myocarditis diagnosis.^38^ However, the median hospital length of stay was 4 to 5 days for both unvaccinated and vaccinated patients, enabling sufficient time for adequate diagnostic procedures and indicative of no difference in disease severity between vaccinated and unvaccinated cases. Deaths were rare, with no deaths of persons younger than 40 years. Our findings in children aged 12 to 15 years were limited to relatively few exposed individuals because vaccination in this age group only recently started in most countries.

Surveillance bias, whereby increased focus and media attention on myocarditis as an adverse event after vaccination^39^ resulted in more subclinical cases being diagnosed, cannot be ruled out. Hence, all studies including data on vaccination and myocarditis after April 25, 2021, are likely prone to this potential surveillance bias. However, in our study, surveillance bias is unlikely to fully explain the differences between the first and second dose, between the 2 mRNA vaccines, and between age groups. Denmark and Norway had lower background incidence rates of myocarditis than Finland and Sweden.

We studied rates of myocarditis after a positive test result for SARS-CoV-2 infection. However, SARS-CoV-2 infection is associated with acute and postacute events other than myocarditis, including hospitalizations, intensive care unit admissions, and death.^40^ The present study showed increased risk of myocarditis after a positive test result for SARS-CoV-2 infection, and the risk was highest in the older age groups, whereas the risk of myocarditis after vaccination was highest in the younger age groups. However, the estimated risk of any outcome after SARS-CoV-2 infection will be dependent on the testing strategy. If only severe COVID-19 cases are tested, the association with other events will be strengthened owing to selection bias. Therefore, to reduce selection bias in our analyses of myocarditis after SARS-CoV-2 infection, we included only the period from August 2020 onward, when testing was widely available in the Nordic countries.

The 2 mRNA vaccines were used in the Nordic countries according to availability during 2021, and supply was limited during the first months of 2021. Furthermore, vaccination was first provided for older adults. The availability has thus varied across age, calendar months, and countries. The background incidence rate of myocarditis fluctuates with infectious disease burden, being typically higher during the fall and winter.^41^ Moreover, differences in lockdown measures affecting the spread of SARS-CoV-2 and other viruses could also affect the background incidence rate in both unvaccinated and vaccinated persons. Most of the younger age groups were vaccinated from July to September 2021, and very few during the spring. However, our supplementary model 3 with adjustment for calendar period resulted in wider CIs but did not substantially change the point estimates.

The observed risks of myocarditis and pericarditis are applicable to the current SARS-CoV-2 pandemic situation in the Nordic countries. In other settings, the background incidence of myocarditis and pericarditis and risks following vaccination may differ. Furthermore, we cannot draw conclusions from the study results to predict myocarditis and pericarditis after a third dose or for children younger than 12 years. We captured all hospitalizations for myocarditis and pericarditis in the Nordic countries during the study period; however, without access to data on clinical measures and diagnostic imaging results, future adjudication must assess how many of these patients fulfill all criteria for a myocarditis diagnosis. Although studies on the long-term prognosis of vaccine-associated cases of myocarditis are lacking and are urgently needed, some evidence suggests that the 28-day risk of death, hospital readmission rates, and development of heart failure appear low, especially in the younger age groups.^34^

## Conclusions

In this cohort study of 23.1 million Nordic residents aged 12 years or older, the risk of myocarditis was higher within 28 days of vaccination with both BNT162b2 and mRNA-1273 compared with being unvaccinated, and higher after the second dose of vaccine than the first dose. The risk was more pronounced after the second dose of mRNA-1273 than after the second dose of BNT162b2, and the risk was highest among males aged 16 to 24 years. Our data are compatible with 4 to 7 excess events within 28 days per 100 000 vaccinees after a second dose of BNT162b2, and 9 to 28 excess events within 28 days per 100 000 vaccinees after a second dose of mRNA-1273. The risk of myocarditis associated with vaccination against SARS-CoV-2 must be balanced against the benefits of these vaccines.

## Notes

Supplement. **eMethods.** Descriptions of Data Sources, Washout Period, Outcome Events, and Ethical Regulations **eTable 1.** Definition of Myocarditis and Pericarditis Outcome and Washout **eTable 2.** Definition of Covariates per Country **eTable 3.** Number of Persons Vaccinated by Country **eTable 4.** Pericarditis Within 28 Days Following a Dose of SARS-CoV-2 Vaccine, According to Sex and Age **eTable 5.** Myocarditis, Pericarditis, and Myocarditis and Pericarditis Combined Within 28 Days Following a Dose of SARS-CoV-2 Vaccine, According to Sex and Age, and Model for Adjustment **eTable 6.** Myocarditis and Pericarditis Combined Within 28 Days Following a Dose of SARS-CoV-2 Vaccine, Boys 12-15 years **eTable 7.** Myocarditis Within 28 Days of a Positive SARS-CoV-2 Test, According to Sex and Age **eTable 8.** Myocarditis Within 7 Days Following a Dose of SARS-CoV-2 Vaccine, According to Sex and Age **eTable 9.** Days From Vaccination to Date of Admission for Vaccinated Myocarditis Cases **eTable 10.** Distribution of Comorbidities for Total Population at Start of Follow-up and for Myocarditis Cases, by Vaccination Status; Nordic Countries Denmark, Finland, Norway, and Sweden Combined **eTable 11.** Mortality and Discharge Outcomes Among Myocarditis Cases **eFigure 1.** Myocarditis in Females Within 28 Days Following SARS-CoV-2 Vaccination **eFigure 2.** Meta-analysis Results of Myocarditis Following COVID-19 Vaccination in 4 Nordic Countries
