# Supplementary material for: SARS-CoV-2 Vaccination and Myocarditis in a Nordic Cohort Study of 23 Million Residents
Source: JAMA Cardiol. 2022 Apr 20;7(6):600–12. doi: 10.1001/jamacardio.2022.0583 (PMC9021987; doi:10.1001/jamacardio.2022.0583)
Supplement: Supplement. — eMethods. Descriptions of Data Sources, Washout Period, Outcome Events, and Ethical Regulations eTable 1. Definition of Myocarditis and Pericarditis Outcome and Washout eTable 2. Definition of Covariates per Country eTable 3. Number of Persons Vaccinated by Country eTable 4. Pericarditis Within 28 Days Following a Dose of SARS-CoV-2 Vaccine, According to Sex and Age eTable 5. Myocarditis, Pericarditis, and Myocarditis and Pericarditis Combined Within 28 Days Following a Dose of SARS-CoV-2 Vaccine, According to Sex and Age, and Model for Adjustment eTable 6. Myocarditis and Pericarditis Combined Within 28 Days Following a Dose of SARS-CoV-2 Vaccine, Boys 12-15 years eTable 7. Myocarditis Within 28 Days of a Positive SARS-CoV-2 Test, According to Sex and Age eTable 8. Myocarditis Within 7 Days Following a Dose of SARS-CoV-2 Vaccine, According to Sex and Age eTable 9. Days From Vaccination to Date of Admission for Vaccinated Myocarditis Cases eTable 10. Distribution of Comorbidities for Total Population at Start of Follow-up and for Myocarditis Cases, by Vaccination Status; Nordic Countries Denmark, Finland, Norway, and Sweden Combined eTable 11. Mortality and Discharge Outcomes Among Myocarditis Cases eFigure 1. Myocarditis in Females Within 28 Days Following SARS-CoV-2 Vaccination eFigure 2. Meta-analysis Results of Myocarditis Following COVID-19 Vaccination in 4 Nordic Countries [file jamacardiol-e220583-s001.pdf]

Supplementary Online Content

Karlstad Ø, Hovi P, Husby A, et al. SARS-CoV-2 vaccination and myocarditis in a Nordic cohort study of 23 million residents. *JAMA Cardiol*. Published online April 20, 2022. doi:10.1001/jamacardio.2022.0583

eMethods. Descriptions of Data Sources, Washout Period, Outcome Events, and Ethical Regulations

eTable 1. Definition of Myocarditis and Pericarditis Outcome and Washout

eTable 2. Definition of Covariates per Country

eTable 3. Number of Persons Vaccinated by Country

eTable 4. Pericarditis Within 28 Days Following a Dose of SARS-CoV-2 Vaccine, According to Sex and Age

eTable 5. Myocarditis, Pericarditis, and Myocarditis and Pericarditis Combined Within 28 Days Following a Dose of SARS-CoV-2 Vaccine, According to Sex and Age, and Model for Adjustment

eTable 6. Myocarditis and Pericarditis Combined Within 28 Days Following a Dose of SARS-CoV-2 Vaccine, Boys 12-15 years

eTable 7. Myocarditis Within 28 Days of a Positive SARS-CoV-2 Test, According to Sex and Age

eTable 8. Myocarditis Within 7 Days Following a Dose of SARS-CoV-2 Vaccine, According to Sex and Age

eTable 9. Days From Vaccination to Date of Admission for Vaccinated Myocarditis Cases

eTable 10. Distribution of Comorbidities for Total Population at Start of Follow-up and for Myocarditis Cases, by Vaccination Status; Nordic Countries Denmark, Finland, Norway, and Sweden Combined

eTable 11. Mortality and Discharge Outcomes Among Myocarditis Cases

eFigure 1. Myocarditis in Females Within 28 Days Following SARS-CoV-2 Vaccination

eFigure 2. Meta-analysis Results of Myocarditis Following COVID-19 Vaccination in 4 Nordic Countries

eReferences

This supplementary material has been provided by the authors to give readers additional information about their work.

© 2022 Karlstad Ø et al. *JAMA Cardiology.*

eMethods. Descriptions of Data Sources, Washout Period, Outcome Events, and Ethical Regulations

Description of data sources in Denmark

The Civil Registration System

The Danish Civil Registrations System (CRS) forms the backbone of the Danish cohort by providing a mandatory unique personal identifier for all permanent residents of Denmark[1]. The system has been used in Denmark since April 2, 1968. The personal identifier is used in all Danish health care services and civil registrations systems and thus allows linkage between registries.

The Danish Vaccination Register

All vaccinations given in Denmark since November 15, 2015, has been mandatorily reported to the Danish Vaccination Register. The information includes personal identifier and date of vaccination. The vaccine products are specified by a unique identifier based on a combination of brand name, substance, formulation, additionally batch number, and dose number (for repeated doses).[2] For the study we also used separate information on governmentally assigned COVID-19 vaccine priority groups (e.g. nursing home residents or health care/social care workers) that was provided on an individual level by the Danish Health Agency to The Danish Health Data Authority.

The Danish National Patient Register

Outcomes and comorbidities were defined by hospital-registered ICD-10 codes from The Danish National Patient Register. The register covers all hospital-contacts in Denmark, with information on the duration of the contact, department of admission, and other hospital characteristics, in addition to the unique personal identifier of the patient[3].

The Danish Microbiology Database (MiBa)

Information on positive PCR tests for SARS-CoV-2 were drawn from The Danish Microbiology Database (MiBa). MiBa contains information on all microbiology samples analysed at Danish departments of microbiology, including information on the freely available SARS-CoV-2 PCR tests the were available regardless of symptoms status throughout the study period. The registry contains information on date of sampling, date of analysis, type of test, interpretation of test, in addition to the unique personal identifier of the patient[4].

Description of data sources in Finland

The National Vaccination Register

All vaccine administrations in Finland, including those against COVID-19 infection, are routinely and automatically transferred to the Primary Health Care Visits Register and extracted to the National Vaccination Register[5]. The information includes personal identifier and date of vaccination. The vaccine products are specified by a unique identifier based on a combination of brand name, substance, formulation, additionally batch number, and dose number (for repeated doses).

National Infectious Diseases Register

The register[6] contains information on notifiable diseases which must be reported by the laboratories and the physician treating the patient, or performing an autopsy, in accordance with the Finnish Communicable Diseases Act. All laboratory-confirmed SARS-CoV-2 infections are recorded in the National Infectious Diseases Register. The register includes personal identifier, date of positive test, and diagnosis of notifiable infectious disease. The register is held by Finnish Institute for Health and Welfare.

National Care Register for Health Care

The register comprises information on all in-hospital care and out-patient specialist care in Finland[7]. The information includes personal identifier, admission and discharge dates, whether hospitalization was planned or acute, codes for discharge diagnoses and surgical procedures, whether discharged as deceased, to own private residence or other health care facilities, type of department, and hospital. It has nationwide coverage regarding in-patient care since 1969 and specialized outpatient care since 1998. Information on primary care is included in another register. During the study period diagnoses were recorded according to the 10th revision of the International Statistical Classification of Diseases and Related Health Problems (ICD-10). ICD-codes for malignancies were included in the data collection. The register is held by Finnish Institute for Health and Welfare. During the entire follow-up period updates from the entire country have had delays of only a couple of weeks. We have no reason to believe that reporting delay or -quality would be dependent on either vaccine status or outcome.

The Finnish Population Information System

The register is an electronic register including personal data of all permanent residents in Finland. It contains information on personal identifier, date of birth, country of birth, place of residence, marital status, date of death, and date of immigration and emigration[8]. The register is held by the Digital and Population Data Services Agency.

Register of Social Assistance

The register contains information on elderly and persons with disability need for social assistance including social rehabilitation[9]. This assistance may be given in nursing homes, people's own homes or other institutions. For this study the register is utilized to identify 24-hour care regardless of its location. The register is held by the Finnish Institute for Health and Welfare.

Statistics on Reimbursements for Medical Expenses

The register contains data that includes that on recipients that have received a right for reimbursements for medications[10]. For such a reimbursement a patient's doctor's statement of the condition is needed and also

an in-house doctor's approval, leading to high specificity in detecting the starting points of relevant diseases. The data includes a specific code for the condition and during the follow-up time period, also ICD-10 coding. The register is held by the Social Insurance Institute

Terhikki Register

The register is a nationwide register containing person level data on rights to act as health care personnel[11].

Description of data sources in Norway

The Norwegian national health registries in this study contain registrations of all contacts with health care services, which are mandatory to report, and linked to reimbursement. Diagnostic codes and dates of contact are registered for each individual with a personal identification number (pin), which is issued to all citizens in Norway at birth or immigration for identification and administrative purposes. The pin enables linkages of individual level information across registries.

The Emergency Preparedness Register for COVID-19

Data in this study were provided through the Emergency preparedness register for COVID-19 ("Beredt C19") administered by the Norwegian Institute of Public Health, according to the Norwegian Health Preparedness Act §2-4 and includes the total population in Norway[12]. This registry was established in 2020 to provide authorities with up-to-date information on prevalence, causal relationships, and consequences of the COVID-19 epidemic in Norway. "Beredt C19" compiles daily updated individual-level data from several registers includes information already collected in the healthcare system, national health registries and administrative registers and is this study we used data from the following sources:

Norwegian Population Register:

The Norwegian Population Register includes date of birth, immigration, emigration and death for all residents of Norway.

The Norwegian Immunisation Register (SYSVAK)

SYSVAK is a register of vaccines in Norwegian vaccination programs, with mandatory registrations of all COVID-19 vaccinations[13]. The information includes personal identifier and date of vaccination. The vaccine products are specified by a unique identifier based on a combination of brand name, substance, formulation.

The Norwegian Patient Registry (NPR)

The Norwegian Patient Registry includes individual level information on all contacts with specialist health-care services[14]. Information registered includes admission and discharge dates, and diagnostic codes during the hospital stay/outpatient contact. These codes are according to the International Classification of Diseases version 10. We used these codes to identify hospitalizations for myocarditis and pericarditis as well as underlying chronic conditions and comorbidities from inpatient or outpatient stays or contacts with private-practicing specialists.

Norwegian Surveillance System for Communicable Diseases (MSIS)

There is mandatory reporting of selected infectious diseases to this National Health register. Reporting of all Covid-19 tests is mandatory, and this register contains date of testing and test results.

State register of employers and employees (NAV AA register)

The Aa Register lists all employment relationships in Norway, and employers and contractors are obliged to report their employees and freelancers in the register via the a-melding (employee and payroll report)[15]. The register provides further information about each employment relationship. Employees are classified according to the Norwegian Standard Classification of Occupations). We obtained data on health care personnel status from the register.

The Norwegian Information System for the Nursing and Care Sector (IPLOS)

IPLOS is a national register containing information on health and care services provided by municipalities in Norway. It is mandatory for municipalities to report to IPLOS for all applicants and recipients of such services. It covers home care service and out-of-hospital institutional care, including short- and long-term nursing home stay. For the current study we used information about nursing-home stays and date of receiving such services[16].

Description of data sources in Sweden

The Swedish data sources are also described in detail elsewhere (Ljung R, Sundström A, Grünewald M, Backman C, Feltelius N, Gedeborg R, Zethelius B. The profile of the COvid-19 VACcination register SAFEty study in Sweden (CoVacSafe-SE). *Ups J Med Sci. 2021 Dec 10;126. doi:10.48101/ujms.v126.8136. eCollection 2021*

The national vaccination register

The national vaccination register contains since Jan 1st, 2021 information on vaccination for COVID-19[17]. The information includes personal identifier and date of vaccination. The vaccine products are specified by a unique identifier based on a combination of brand name, substance, formulation, batch number, and dose number (for repeated doses). The completeness of registration within the child immunization program is high, where 98,4% of children had at least one vaccination recorded, however, completeness of registration of vaccination for COVID-19 has not been published. The register is held by the Public Health Agency of Sweden.

Register on surveillance of notifiable communicable diseases (SmiNet)

SmiNet contains information on notifiable diseases which must be reported by the laboratories and the physician treating the patient, or performing an autopsy, in accordance with the Swedish Communicable Diseases Act[18]. SmiNet includes personal identifier, date of disease occurrence, date of testing, date of positive test, and diagnosis of notifiable infectious disease. The register is held by the Public Health Agency of Sweden.

The Swedish patient register

The Swedish patient register comprises information on all in-hospital care and out-patient specialist care in Sweden[19,20]. The information includes personal identifier, admission and discharge dates, whether hospitalisation was planned or acute, codes for discharge diagnoses and surgical procedures, whether discharged as deceased, to own private residence or other health care facilities, type of department, and hospital. It has nation-wide coverage regarding in-patient care since 1987 and specialized outpatient care since 2001. The in-hospital part of the register is a discharge register; hence, the admission is reported at time of discharge. There is no information on admission diagnoses. There is no information on primary care. During the study period diagnoses were recorded according to the Swedish clinical modification of the 10th revision of the International Statistical Classification of Diseases and Related Health Problems (ICD-10-SE). Monthly updates, but there is a lag in reporting to the register, for the last four weeks before end-of-follow up Oct 3, 2021, the completeness compared to previous years was 91%, 81%, 75%, and 58% respectively. We have no reason to believe that reporting from health care providers and county councils to the National Board of Health and Welfare are dependent on either vaccine status or outcome. The register is held by the National Board of Health and Welfare.

The Swedish Cancer Register

The Swedish Cancer Register was set up in 1958[21,22]. Every clinician, pathologist and cytologist in Sweden must report a new primary malignancy. The Cancer Register includes primary malignancies and certain benign tumours and precancerous lesions. In comparison to the National Patient Register, the proportion of nonreporting to the National Cancer Registry was estimated to be 3.7% in 1998. The register is held by the National Board of Health and Welfare.

The Swedish Prescribed Drug Register

The Swedish Prescribed Drug Register contains details of all the prescriptions dispensed in Sweden since July 1, 2005[23,24]. It is updated monthly with around 100 million prescriptions dispensed each year. It covers the entire Swedish population and includes information on unique personal identifier of the patient, age, sex, place of residence, and prescription information on substance, brand name, formulation and package dispensed amount, dosage (in free text) and unique expenditure and reimbursement, date of prescribing and dispensing, practice that has issued the prescription, and prescriber's profession. Drugs are identified by a unique identifier for each specific combination of brand name, substance, formulation, and package. Additionally, all drugs are classified according to the Anatomic Therapeutic Chemical Classification System (ATC). The register only includes filled prescriptions, not medicines sold over the counter, nor medicines administered directly by health-care personnel without prescription. The register is held by the National Board of Health and Welfare.

The Swedish cause of death register

The Swedish cause of death register contains information on personal identifier, sex, date and country of birth, place of residence at time of death, date and underlying cause of death and contributing causes of death, place of death (hospital, nursing home or assistant living, private residence, or other/unknown), autopsy type, and whether the deceased had undergone a surgical procedure within 4 weeks prior, and whether the death occurred abroad. Data is available for all deaths of Swedish residents since 1952, and it has a 99.2% completeness of causes of death[25]. While some registered causes of death are non-specific 96% of deaths have a specific underlying cause of death recorded. The register is updated annually but during the pandemic all deceased with an underlying cause of death of covid-19 have been continuously registered. Hence, cause of death due to covid-19 is available with only some weeks delay, whereas other causes of death usually are finalized during late spring the following year. Date of death, without information on underlying cause of death, is available with a lag time of around two weeks. The register is held by the National Board of Health and Welfare.

The Total Population Register

The Total Population Register contains information on personal identifier, date of birth, country of birth, place of residence, marital status, date of death, and date of immigration and emigration[26]. The register is held by Statistics Sweden.

Register on persons in nursing homes

The register contains information on elderly and persons with physical, psychiatric or intellectual disabilities, given nursing care in nursing homes, their own homes or other institutions[27]. For this study the register is solely used to define elderly aged 65 years and older living in nursing homes. The register is held by the National Board of Health and Welfare.

The Longitudinal integrated database for health insurance and labour market studies

The longitudinal integrated database for health insurance and labour market studies (LISA) contains information on a wide range of socioeconomic factors. Information on healthcare worker occupation was retrieved from the register[28]. The register is held by Statistics Sweden.

Formulas

IR= Incidence rate

IRR= Incidence rate ratio

PYR=person-years

IR=events/PYR

Exrate= IR-IR/IRR

Excess events in 28 days per 100 000: (1-exp(-exrate*28/365)) *100000

Description of washout period and case definition of outcome events

Individuals with a myocarditis or pericarditis diagnosis (as defined under secondary study outcomes) between January 1, 2017, and December 27, 2020, were excluded from all analyses. I.e., for the analyses with myocarditis as outcome both previous myocarditis and pericarditis are used to exclude individuals. The same applies for analyses of pericarditis (persons with previous care for either myocarditis or pericarditis are excluded). In total, 20,211 were excluded, whereof 3,706 from Denmark, 3164 from Finland, 4,864 from Norway, and 8,477 from Sweden.

During follow-up (after December 27, 2020) we regard inpatient as outcome events (refer to outcome definitions). If a patient has an outpatient event of either myocarditis or pericarditis, this is regarded as a censoring event (not outcome event, note: Finland made no censoring on this reason), The exception is if an outpatient-visit (with myocarditis or pericarditis) or an in-patient admission discharge date occurs (with myocarditis or pericarditis) within 7 days prior of an in-patient admission under study as outcome event. Then that outpatient visit is regarded as being within the same care episode. This is regardless of whether the outpatient visit was diagnosed as myocarditis or pericarditis. In that case, the date of the outcome event is recoded (moved back) to the date of the outpatient event. Furthermore, if there are any myocarditis codes registered within the same care episode then the episode is always regarded only as myocarditis outcome regardless of whether pericarditis is also registered during that episode.

Descriptions of Ethical Regulations

Denmark

The Danish study was conducted using administrative register data. According to Danish law, ethics approval is not required for such research.

Finland

The Finnish study is a part of vaccine surveillance work, which is one of the duties of the Finnish Institute for Health and Welfare (THL). For this work, the institute is obliged to utilize all available data, including register data, to investigate potential harmful effects of the vaccines[29]. Consent to participate was not applicable as this is a register-based study.

Norway

The Norwegian study was approved by the Norwegian Regional Committee for tHealth Research Ethics South East (REK Sør-Øst A, ref 122745), and has conformed to the principles embodied in the Declaration of Helsinki. The emergency preparedness register was established according to the Health Preparedness Act §2-4. Consent to participate was not applicable as this is a register-based study.

Sweden

The Swedish study is approved by the Swedish Ethical Review Authority (2020-06859, 2021-02186) and has conformed to the principles embodied in the Declaration of Helsinki. Consent to participate is not applicable as this is a register-based study.

eTable 1. Definition of Myocarditis and Pericarditis Outcome and Washout

Item	Implementation used per country
**Main outcomes - inpatient stay**	
• Myocarditis (ICD-10 codes: I400 I401 I408 I409 I411 I418 I514).<br>• Pericarditis (ICD-10 codes: I300 I301 I308 I309 I328)<br>One or more records with primary or secondary discharge diagnosis code.<br>Target definition is inpatient stay.<br>Time period: from December 27, 2020 onwards.	Denmark: Hospitalization lasting more than 24 hours.
	Finland: Non-scheduled inpatient hospital care.
	Norway: Inpatient hospital care with overnight stay.
	Sweden: Inpatient hospital care (97.2% of myocarditis cases had overnight stay, 94.2% pericarditis cases had overnight stay).
**Secondary outcome**	
Myocarditis or pericarditis (combined)<br>ICD-10 codes: I400 I401 I408 I409 I411 I418 I514 I300 I301 I308 I309 I328.<br>One or more records with primary or secondary discharge diagnosis code.<br>Target definition is either inpatient or outpatient care.<br>Time period: from December 27, 2020 onwards.	Denmark: Any hospital-registered diagnosis of myocarditis or pericarditis.
	Finland: Non-scheduled inpatient hospital care or specialized hospital emergency room visit (Also cases with B332, Carditis, included)
	Norway: Inpatient or outpatient hospital care regardless of length of stay.
	Sweden: Inpatient hospital care regardless of length of stay, or specialized outpatient care (both hospital based and private practitioner).
**Washout for prior outcome**	
1+ record with primary/secondary diagnosis for either myocarditis or pericarditis during January 1, 2017 – December 27, 2020.<br>ICD-10 codes: I400 I401 I408 I409 I411 I418 I514 I300 I301 I308 I309 I328	Denmark: Same as secondary outcome.
	Finland: Same as secondary outcome.
	Norway: Same as secondary outcome.
	Sweden: Same as secondary outcome.
End of follow up	Denmark: October 5, 2021.
	Norway: October 5, 2021.
	Finland: October 5, 2021.
	Sweden: October 3, 2021.

eTable 2. Definition of Covariates per Country

Table S2A. Covariate information and data sources used in Denmark.		
**Covariate**	**Data source[a]**	**Definitions used**
**Vaccine priority groups:**	DHA	Vaccine priority groups used for analysis:<br>- Nursing home residents.<br>- Individuals aged 65 years or older who receive domestic help.<br>- Health and social care workers.<br>- Vulnerable individuals.<br>- Relatives in close contact with individuals at increased risk of severe disease.<br>- Individuals prioritized by age alone.
**Comorbidity groups: (binary)**	DNPR	**ICD-10 codes (primary diagnoses, regardless of length of hospitalisation)**
Chronic pulmonary disease	DNPR	J40-J44, J45-J46, J47, J60–J67, J68.4, J70.1, J70.3, J84.1, J92.0, J96.1, J98.2, J98.3
Cardiovascular conditions and Diabetes	DNPR	I20-I23<br>I11.0, I13.0, I13.2, I42.0, I42.6, I42.7, I42.8, I42.9, I50.0, I50.1, I50.2, I50.3, I50.8, I50.9<br>E10.0, E10.1, E10.2-E10.8, E10.9, E11.0, E11.1, E11.2-E11.8, E11.9, I48
Autoimmunity-related conditions	DNPR	K50.x, K51.x<br>M32.x, M05.x-M06.x, E05.0, E06.3, G35.x, L40.x, E27.1, E27.2, G12.2G, M45.x, M08.1, K90.0, M33.x, L52.x, G61.0, D59.0-D59.1, D69.0, D69.3, M08.x, L93.x, G70.0, D51.0, L12.x, M31.3, M30.0, K74.3, I00.x-01.x, D86.x, M34.x, M31.5-M31.6, L80.x, M35.x
Malignancy	DNPR	C00–C75, C76-C80, C81-C85, C88, C90, C91-95, C96
Moderate to severe renal disease	DNPR	I12, I13, N00–N05, N07, N11, N14, N17–N19, Q61

**SARS-CoV-2 infection:**	MiBa	Positive SARS-CoV-2 PCR test (prior to start of follow-up on December 27, 2020)

[a] Data sources in Denmark: The Danish Health Agency (DHA), The Danish National Patient Register (DNPR), The Danish Microbiology Database (MiBa).

eTable 2B. Covariate Information and Data Sources Used in Finland

Covariate	Data source[a]	Definitions used
**Vaccine priority groups:**	Terhikki<br><br>Register of Social assistance	Healthcare worker (status per December 27, 2020).<br><br>Nursing home resident (status per December 27, 2020).
**Comorbidity groups: (binary)**		
Chronic pulmonary disease	th, SII	(ICD-10): J41, J42, J43, J44, J45, J46, J47
Cardiovascular conditions and Diabetes		The conditions listed are included in the model as one 1/0 combined variable
	th, SII	(SII:) 206, (ICD-10:) I20, I21, I22, I23, I24, I25
	th, SII	(SII:) 201, (ICD-10:) I10, I130, I132, I150
	th, ah, purch	(ICD-10) E11, E13, E14, (ATC:) A10A (ICPC2:) T90
Autoimmunity-related conditions	th, SII	(SII:) 132,134,202, 208 (ICD-10:) D86, L40, M02, M05, M06, M07, M139, M45, M460, M461, M469, M941, K50, K51
Malignancy	th, SII	(SII:) 117, 115, 116, 128, 130, (ICD-10:) C00 – C97, D051, D39
Moderate to severe renal disease	th, SII	(ICD-10:) N03, N18, I12, I13, N00, N01, N02, N03, N04, N05, N07, N08, N11, N14, N18, N19, E102, E112, E142
**SARS-CoV-2 infection:**	TTR	Positive SARS-CoV-2 PCR test (prior to start of follow-up on December 27, 2020)

[a] Data sources in Finland: Terhikki register, Register of Social assistance, Care register for Health Care (th), Statistics on reimbursements for medical expenses, Social Insurance Institute (SII), National Infectious Diseases Register (TTR).

eTable 2C. Covariate Information and Data Sources Used in Norway

Covariate	Data source[a]	Definitions used
**Vaccine priority groups (binary)**	AA register<br><br>IPLOS	Healthcare worker (status per December 27, 2020).<br>Nursing home resident (status per December 27, 2020).
**Comorbidity groups (binary)**	NPR	**Any records with ICD-10 codes as primary/secondary diagnosis from inpatient stay or outpatient contact in hospital or from private-practicing specialists, January 1, 2017 – December 27, 2020)**
Chronic pulmonary disease	NPR	J41-J44, J45-J46, J47 J701 J703 J84 J98 E84
Cardiovascular conditions and diabetes	NPR	I20-I23, I25-I28, I110, I130, I132, I1420, I426-I429, I50, I05-I09, I33-I39, I48, E10 E11 E12 E13 E14
Autoimmunity-related conditions	NPR	G35, M05-M09, M13-M14, K50-K51
Malignancy	NPR	C00-C96
Moderate to severe renal disease	NPR	I12-I13, N00-N05, N07, N11, N14, N17-N19, Q61
**SARS-CoV-2 infection**	MSIS	Positive SARS-CoV-2 PCR test (prior to start of follow-up on December 27, 2020)

[a] Data sources in Norway: Norwegian Patient Register (NPR), Norwegian Surveillance System for Communicable Diseases (MSIS), Norwegian Information System for the Nursing and Care Sector (IPLOS), State register of employers and employees (AA register), Reverse transcription polymerase chain reaction (PCR).

eTable 2D. Covariate Information and Data Sources Used in Sweden

Covariate	Data source[a]	Definitions used
**Vaccine priority groups:**		
	LISA	Healthcare worker (status per October 2018)
	SOL	Nursing home resident (status per December 31, 2020).
**Comorbidity groups: (binary)**		**Any records with ICD-10 codes as primary/secondary diagnosis from inpatient stay or outpatient contact in hospital or from private-practicing specialists, January 1, 2017 – December 27, 2020)**
Chronic pulmonary disease	NPR	J41 J42 J43 J44 J45 J46 J47 J84 J98 E84
Cardiovascular conditions and diabetes	NPR, SPDR	I05 I06 I07 I08 I09 I110 I2 I34 I35 I36 I37 I39 I42 I43 I46 I48 I49 I50 E10-E14<br>ATC: A10 (at least two filled prescriptions during 2020, before December 27, 2020)
Autoimmunity-related conditions	NPR	D86 G35 K50 K51 L40 M05 M06 M07 M08 M09 M13 M14 M45
Malignancy	NPR, CAN	C0 C1 C2 C3 C4 C5 C6 C7 C8 C9 D45 D46 D47<br>(CAN from 2017-2019, NPR for 2020)
Moderate to severe renal disease	NPR	I12 I13 N00 N01 N02 N03 N04 N05 N07 N11 N14 N17 N18 N19 Q61
**SARS-CoV-2 infection:**	SmiNet	Positive SARS-CoV-2 PCR test (prior to start of follow-up on December 27, 2020)

[a] Data sources in Sweden: The longitudinal integrated database for health insurance and labour market studies (LISA), Register on persons in nursing homes (SOL), National Patient Register (NPR), Swedish Prescribed Drug Register (SPDR), Cancer Register (CAN), Register on surveillance of notifiable communicable diseases (SmiNet), Reverse transcription polymerase chain reaction (PCR).

eTable 3. Number of Persons Vaccinated by Country

eTable 3A. Men and Women Combined Contributing to Unexposed and Exposed Person-time by Vaccine Type and Vaccine schedule. Vaccination from December 27, 2020, to October 5, 2021. Denmark.

	Age group				
**Denmark**	**12+**	**12-15**	**16-24**	**25-39**	**40+**
	**N**	**N**	**N**	**N**	**N**
**Population at start-of-follow-up**	4,955,483	264,898	599,287	1,004,908	3,086,390
**Unvaccinated by end-of-follow-up**	726,566	99,454	113,742	265,162	248,208
**Vaccine schedule**					
**At least first dose AZD1222**	146,030	≤5	10,932	39,208	95,887
Only first dose AZD1222	145,462	≤5	10,892	39,061	95,506
AZD1222/AZD1222	568		40	147	381
**At least first dose BNT162B2**	3,571,191	164,880	453,949	437,182	2,515,180
Only first dose BNT162b2	66,843	11,161	15,840	16,628	23,214
BNT162b2/BNT162b2	3,504,348	153,719	438,109	420,554	2,491,966
**At least first dose mRNA-1273**	511,696	561	20,664	263,356	227,115
Only first dose mRNA-1273	16,185	176	1258	12,133	2618
mRNA-1273/BNT162b2					
mRNA-1273/mRNA-1273	495,511	385	19,406	251,223	224,497

eTable 3B. Men and Women Combined Contributing to Unexposed and Exposed Person-time by Vaccine Type and Vaccine schedule. Vaccination from December 27, 2020, to October 5, 2021. Finland.					
	**Age group**				
**Finland**	**12+**	**12-15**	**16-24**	**25-39**	**40+**
	**N**	**N**	**N**	**N**	**N**
**Population at start-of-follow-up**	4,943,536	249,833	545,949	1,079,655	3,068,099
**Unvaccinated by end-of-follow-up**	806,786	81,101	134,342	270,566	320,777
**Vaccine schedule**					
**At least first dose AZD1222**	361,182	23	3076	13,858	344,225
Only first dose AZD1222	6660	13	138	463	6046
AZD1222/AZD1222	189,783	≤5	23	72	189,687
AZD1222/BNT162b2	135,001	≤5	2204	10476	122,320
AZD1222/mRNA-1273	29,738	8	711	2847	26,172
**At least first dose BNT162B2**	3,247,962	85,945	345,744	689,450	2126,823
Only first dose BNT162b2	402,074	67,281	98,457	109,447	126,889
BNT162b2/BNT162b2	2,795,208	17,571	233,345	563,174	1,981,118
BNT162b2/mRNA-1273	49,340	1092	13,916	16,746	17,586
BNT162B2/AZD1222	1340	≤5	26	83	1230
**At least first dose mRNA-1273**	527,513	82,764	62,777	105,736	276,236
Only first dose mRNA-1273	175,660	60,633	35,817	41,898	37,312
mRNA-1273/BNT162b2	12,897	620	2292	3424	6561
mRNA-1273/mRNA-1273	338,757	21,507	24,653	60,390	232,207
mRNA-1273/AZD1222	199	≤5	15	24	156
**Other vaccinations**	93	0	10	45	38

**eTable 3C. Men and Women Combined Contributing to Unexposed and Exposed Person-time by Vaccine Type and Vaccine schedule. Vaccination from December 27, 2020, to October 5, 2021. Norway**					
	**Age group**				
**Norway**	**12+**	**12-15**	**16-24**	**25-39**	**40+**
	**N**	**N**	**N**	**N**	**N**
**Population at start-of-follow-up**	4,575,079	255,424	566,952	1,033,731	2,718,972
**Unvaccinated by end-of-follow-up**	621,986	103,185	89,615	186,369	242,817
**Vaccine schedule**					
**At least first dose AZD1222**	-[a]	-[a]	11,058	38,429	84,453
Only first dose AZD1222	4 005	66	392	1590	1957
AZD1222/AZD1222	495	0	63	137	295
AZD1222/BNT162b2	-[a]	≤5	10,402	35,938	80,277
AZD1222/mRNA-1273	2889	0	201	764	1924
**At least first dose BNT162B2**	3,326,428	152,012	411,721	643,978	2,118,717
Only first dose BNT162b2	365,589	151,361	116,045	46,698	51,485
BNT162b2/BNT162b2	2,412,340	628	185,653	369,175	1,856,884
BNT162b2/mRNA-1273	548,499	23	110,023	228,105	210,348
**At least first dose mRNA-1273**	490,608	160	54,404	163,976	272,068
Only first dose mRNA-1273	64,130	145	13,385	32,444	18,156
mRNA-1273/BNT162b2	39,415	7	7334	19,120	12,954
mRNA-1273/mRNA-1273	387,063	8	33,685	112,412	240,958
**Other vaccinations**	-[a]	≤5 -[a]	154	979	917

[a] Numbers removed so cells with count ≤5 cannot be recalculated from other cell counts (data privacy regulations).

**eTable 3D. Men and Women Combined Contributing to Unexposed and Exposed Person-time by Vaccine Type and Vaccine schedule. Vaccination from December 27, 2020, to October 3, 2021. Sweden.**					
	**Age group**				
**Sweden**	**12+**	**12-15**	**16-24**	**25-39**	**40+**
	**N**	**N**	**N**	**N**	**N**
**Population at start-of-follow-up**	8,648,424	467,849	963,370	1,927,870	5,289,335
**Unvaccinated by end-of-follow-up**	2,153,116	466,513	327,864	587,550	771,189
**Vaccine schedule**					
**At least first dose AZD1222**	715,238	≤5	13,354	605,42	641,340
Only first dose AZD1222	22,320	≤5	1061	4126	17,132
AZD1222/AZD1222	574,809		4498	22,364	547,947
AZD1222/BNT162b2	101,223	≤5	6858	28,750	65,614
AZD1222/mRNA-1273	16,886		937	5302	10,647
**At least first dose BNT162B2**	4,919,004	1078	463,130	1,047,324	3,407,472
Only first dose BNT162b2	297,049	548	86,083	98,343	112,075
BNT162b2/BNT162b2	4,604,061	530	372,483	941,213	3,289,835
BNT162b2/mRNA-1273	17,280		4556	7749	4975
BNT162b2/AZD1222	614		8	19	587
**At least first dose mRNA-1273**	861,053	256	159,020	232,450	469,327
Only first dose mRNA-1273	115,953	199	45,541	40,954	29,259
mRNA-1273/BNT162b2	5770	≤5	1676	1737	2355
mRNA-1273/mRNA-1273	739,263	55	111,801	189,756	437,651
mRNA-1273/AZD1222	67		≤5	≤5	62
**Other vaccinations**	13		≤5	≤5	7

eTable 4. Pericarditis Within 28 Days Following a Dose of SARS-CoV-2 Vaccine, According to Sex and Age

Incidence Rate Ratios (IRR) and Excess Cases in 28 Days per 100,000 Vaccinees.

Subgroup, exposure	Events	1000 PYR	IR per 1000 PYR	IRR (95%CI)	Excess in 28 days per 100,000 (95%CI)
**Males, 12+**					
Unvaccinated	547	5340.6	0.102	1 (ref)	0 (ref)
AZD1222	8	43	0.186	1.25 (0.61-2.56)	0.29 (-0.55-1.13)
AZD1222/AZD1222	≤5	29.2		0.86 (0.32-2.34)	-0.17 (-1.4-1.06)
BNT162b2	93	560.9	0.166	1.29 (0.96-1.74)	0.29 (-0.01-0.58)
BNT162b2/BNT162b2	88	495	0.178	1.38 (1.1-1.74)	0.38 (0.14-0.62)
BNT162b2/mRNA-1273	10	23.7	0.421	2.95 (1.55-5.59)	2.14 (0.64-3.64)
mRNA-1273	10	93.2	0.107	1.11 (0.59-2.07)	0.08 (-0.39-0.55)
mRNA-1273/mRNA-1273	26	72.3	0.360	2.99 (2.02-4.44)	1.84 (1.04-2.63)
**Males, 16-24**					
Unvaccinated	58	794.6	0.073	1 (ref)	0 (ref)
AZD1222	0	0.7	...	...	...
AZD1222/AZD1222	0	0.1			
BNT162b2	≤5	63.9		1.19 (0.42-3.34)	0.08 (-0.35-0.5)
BNT162b2/BNT162b2	9	41.5	0.217	2.85 (1.38-5.86)	1.08 (0.26-1.9)
BNT162b2/mRNA-1273	≤5	4.6		6.36 (1.85-21.87)	4.21 (-0.65-9.07)
mRNA-1273	≤5	11.5		2.81 (0.68-11.66)	0.86 (-0.51-2.24)
mRNA-1273/mRNA-1273	6	5.8	1.034	14.78 (6.29-34.77)	7.39 (1.46-13.32)
**Males, 25-39**					
Unvaccinated	110	1440.6	0.076	1 (ref)	0 (ref)
AZD1222	0	3.1	...	...	...
AZD1222/AZD1222	0	0.5	...	...	...
BNT162b2	17	109.2	0.156	2.33 (1.34-4.07)	0.68 (0.25-1.11)
BNT162b2/BNT162b2	18	83.9	0.215	2.95 (1.78-4.88)	1.09 (0.51-1.66)
BNT162b2/mRNA-1273	≤5	9.7	...	4.33 (1.71-10.98)	3.03 (0.24-5.83)
mRNA-1273	≤5	30.6	...	2.29 (0.92-5.66)	0.71 (-0.09-1.5)
mRNA-1273/mRNA-1273	7	23	0.305	4.35 (1.97-9.58)	1.8 (0.4-3.2)

Subgroup, exposure	Events	1000 PYR	IR per 1000 PYR	IRR (95%CI)	Excess in 28 days per 100,000 (95%CI)
**Males, 40+**					
Unvaccinated	373	2657.6	0.140	1 (ref)	0 (ref)
AZD1222	8	39.3	0.204	1.28 (0.63-2.62)	0.34 (-0.56-1.25)
AZD1222/AZD1222	≤5	28.6	…	0.83 (0.31-2.26)	-0.22 (-1.53-1.09)
BNT162b2	72	375.8	0.192	1.26 (0.98-1.62)	0.3 (0-0.61)
BNT162b2/BNT162b2	61	363.6	0.168	1.09 (0.83-1.44)	0.11 (-0.21-0.43)
BNT162b2/mRNA-1273	≤5	9.4	…	1.32 (0.32-5.35)	0.39 (-1.43-2.22)
mRNA-1273	≤5	48	…	0.5 (0.16-1.57)	-0.47 (-1.68-0.74)
mRNA-1273/mRNA-1273	13	43.3	0.300	2.42 (1.39-4.22)	1.35 (0.45-2.26)
**Females, 12+**					
Unvaccinated	237	4942.2	0.048	1 (ref)	0 (ref)
AZD1222	6	64.1	0.094	2.04 (0.76-5.46)	0.37 (-0.09-0.82)
AZD1222/AZD1222	≤5	31.6	…	0.96 (0.23-3.93)	-0.02 (-0.74-0.7)
BNT162b2	43	572.3	0.075	1.25 (0.9-1.74)	0.12 (-0.04-0.27)
BNT162b2/BNT162b2	43	522.7	0.082	1.47 (1.05-2.05)	0.2 (0.04-0.36)
BNT162b2/mRNA-1273	6	19.1	0.315	6.06 (2.6-14.12)	2.02 (0.37-3.66)
mRNA-1273	12	90	0.133	2.88 (1.61-5.16)	0.67 (0.24-1.1)
mRNA-1273/mRNA-1273	14	71.6	0.196	3.41 (1.98-5.87)	1.06 (0.46-1.66)
**Females, 16-24**					
Unvaccinated	17	707.1	0.024	1 (ref)	0 (ref)
AZD1222	0	2.4	…	…	…
AZD1222/AZD1222	0	0.3			
BNT162b2	≤5	63.2		2.47 (0.3-20.58)	0.14 (-0.14-0.43)
BNT162b2/BNT162b2	≤5	43.9		3.43 (0.41-28.65)	0.12 (-0.14-0.39)
BNT162b2/mRNA-1273	≤5	4		11.81 (2.34-59.45)	3.48 (-1.37-8.32)
mRNA-1273	≤5	10.7		23.15 (5.79-92.58)	2.05 (-0.27-4.38)
mRNA-1273/mRNA-1273	≤5	6		28.31 (7.7-104.13)	4.94 (0.09-9.78)
**Females, 25-39**					
Unvaccinated	23	1269.7	0.018	1 (ref)	0 (ref)
AZD1222	0	8.8	…	…	…
AZD1222/AZD1222	0	1.3	…	…	…
BNT162b2	≤5	105		2.35 (0.65-8.42)	0.13 (-0.06-0.31)

Subgroup, exposure	Events	1000 PYR	IR per 1000 PYR	IRR (95%CI)	Excess in 28 days per 100,000 (95%CI)
BNT162b2/BNT162b2	≤5	85		3.34 (1.1-10.11)	0.25 (-0.02-0.53)
BNT162b2/mRNA-1273	≤5	7.5		23.21 (5.52-97.68)	2.95 (-0.39-6.28)
mRNA-1273	≤5	27.7		7.83 (1.69-36.35)	0.48 (-0.2-1.16)
mRNA-1273/mRNA-1273	≤5	21		17.02 (4.85-59.78)	1.03 (-0.14-2.2)
**Females, 40+**					
Unvaccinated	194	2541.6	0.076	1 (ref)	0 (ref)
AZD1222	6	52.9	0.113	2.43 (0.67-8.87)	0.51 (-0.11-1.13)
AZD1222/AZD1222	≤5	30		0.91 (0.22-3.75)	-0.05 (-0.85-0.75)
BNT162b2	38	392.5	0.097	1.22 (0.86-1.74)	0.13 (-0.08-0.35)
BNT162b2/BNT162b2	38	388.1	0.098	1.39 (0.97-1.99)	0.21 (0.01-0.41)
BNT162b2/mRNA-1273	≤5	7.5		1.61 (0.22-11.68)	0.38 (-1.08-1.85)
mRNA-1273	7	48.5	0.144	2.1 (0.99-4.49)	0.58 (-0.01-1.17)
mRNA-1273/mRNA-1273	7	44.4	0.157	2.21 (1.04-4.74)	0.66 (0.02-1.31)

Abbreviations: PYR follow-up time in person years, IR crude incidence rate, IRR adjusted incidence rate ratio (Model 2: Adjusted for age group, sex, previous SARS-CoV-2 infection, healthcare worker, nursing home resident, comorbidity variables).

eTable 5. Myocarditis, Pericarditis, and Myocarditis and Pericarditis Combined Within 28 Days Following a Dose of SARS-CoV-2 Vaccine, According to Sex and Age, and Model for Adjustment

Excess Events in 28 Days per 100,000 Vaccinees.

	Excess events in 28 days per 100,000 (95% CI)		
**Subgroup, exposure**	**Myocarditis**	**Pericarditis**	**Myocarditis and Pericarditis combined**
**Both, 12+, BNT162b2**			
Model 2	0.19 (0.08-0.31)	0.19 (-0.01-0.38)	0.47 (0.26-0.69)
Model 3	0.12 (-0.01-0.25)	0.23 (0.03-0.43)	0.43 (0.18-0.69)
**Both, 12+, BNT162b2/BNT162b2**			
Model 2	0.37 (0.25-0.49)	0.24 (0.07-0.41)	0.84 (0.61-1.07)
Model 3	0.22 (0.07-0.37)	0.27 (0.07-0.46)	0.67 (0.33-1.00)
**Both, 12+, BNT162b2/mRNA-1273**			
Model 2	6.34 (4.32-8.36)	2.08 (0.99-3.18)	11.24 (8.61-13.87)
Model 3	6.25 (4.25-8.25)	2.14 (1.01-3.26)	11.16 (8.53-13.79)
**Both, 12+, mRNA-1273**			
Model 2	0.09 (-0.2-0.37)	0.36 (0.07-0.64)	0.63 (0.17-1.09)
Model 3	-0.03 (-0.38-0.31)	0.39 (0.11-0.67)	0.53 (0.05-1.02)
**Both, 12+, mRNA-1273/mRNA-1273**			
Model 2	2.71 (2.01-3.42)	1.45 (0.95-1.95)	4.84 (3.92-5.75)
Model 3	2.55 (1.86-3.24)	1.47 (0.96-1.98)	4.64 (3.74-5.54)
**Both, 16-24, BNT162b2**			
Model 2	0.81 (0.35-1.26)	0.12 (-0.11-0.35)	1.27 (0.66-1.88)
Model 3	0.64 (0.13-1.15)	0.14 (-0.1-0.39)	1.01 (0.29-1.72)
**Both, 16-24, BNT162b2/BNT162b2**			
Model 2	2.99 (2.04-3.93)	0.51 (0.10-0.92)	4.55 (3.40-5.71)
Model 3	2.89 (1.81-3.97)	0.55 (0.10-0.99)	4.3 (2.97-5.62)
**Both, 16-24, BNT162b2/mRNA-1273**			
Model 2	16.33 (8.98-23.68)	3.86 (0.43-7.3)	24.29 (15.43-33.15)
Model 3	16.47 (9.05-23.89)	3.99 (0.44-7.54)	24.61 (15.62-33.59)
**Both, 16-24, mRNA-1273**			
Model 2	0.84 (-0.16-1.84)	1.41 (0.14-2.68)	2.4 (0.75-4.06)
Model 3	0.83 (-0.25-1.9)	1.53 (0.17-2.88)	2.37 (0.7-4.04)

	Excess events in 28 days per 100,000 (95% CI)		
**Subgroup, exposure**	**Myocarditis**	**Pericarditis**	**Myocarditis and Pericarditis combined**
**Both, 16-24, mRNA-1273/mRNA-1273**			
Model 2	8.88 (4.36-13.4)	6.15 (2.33-9.97)	16.71 (10.46-22.96)
Model 3	8.96 (4.35-13.58)	6.18 (2.34-10.02)	16.57 (10.32-22.83)
**Both, 25-39, BNT162b2**			
Model 2	0.29 (0.02-0.57)	0.37 (0.08-0.66)	0.70 (0.07-1.34)
Model 3	0.11 (-0.23-0.45)	0.40 (0.14-0.65)	0.59 (-0.08-1.27)
**Both, 25-39, BNT162b2/BNT162b2**			
Model 2	0.40 (0.11-0.7)	0.64 (0.32-0.97)	1.44 (0.81-2.07)
Model 3	0.20 (-0.19-0.58)	0.64 (0.31-0.97)	1.28 (0.61-1.96)
**Both, 25-39, BNT162b2/mRNA-1273**			
Model 2	6.33 (3.12-9.54)	3.00 (0.88-5.12)	13.01 (8.47-17.54)
Model 3	6.19 (3.04-9.35)	2.94 (0.82-5.06)	12.86 (8.35-17.38)
**Both, 25-39, mRNA-1273**			
Model 2	0 (-0.46-0.45)	0.59 (0.09-1.1)	1.23 (0.44-2.01)
Model 3	-0.12 (-0.75-0.5)	0.54 (0.03-1.06)	1.05 (0.2-1.89)
**Both, 25-39, mRNA-1273/mRNA-1273**			
Model 2	4.62 (2.92-6.31)	1.39 (0.5-2.28)	7.47 (5.37-9.57)
Model 3	4.40 (2.75-6.04)	1.38 (0.48-2.28)	7.29 (5.21-9.38)
**Males, 12+, BNT162b2**			
Model 2	0.27 (0.09-0.46)	0.29 (-0.01-0.58)	0.80 (0.48-1.13)
Model 3	0.13 (-0.1-0.35)	0.34 (0.02-0.66)	0.70 (0.31-1.09)
**Males, 12+, BNT162b2/BNT162b2**			
Model 2	0.67 (0.46-0.88)	0.38 (0.14-0.62)	1.39 (1.04-1.74)
Model 3	0.42 (0.16-0.67)	0.42 (0.11-0.72)	1.10 (0.68-1.51)
**Males, 12+, BNT162b2/mRNA-1273**			
Model 2	10.34 (6.86-13.83)	2.14 (0.64-3.64)	16.18 (11.94-20.43)
Model 3	10.21 (6.75-13.66)	2.23 (0.68-3.79)	16.13 (11.87-20.39)
**Males, 12+, mRNA-1273**			
Model 2	0.33 (-0.11-0.78)	0.08 (-0.39-0.55)	0.36 (-0.41-1.14)
Model 3	0.12 (-0.42-0.66)	0.11 (-0.35-0.57)	0.14 (-0.72-1)

© 2022 Karlstad Ø et al. *JAMA Cardiology.*

	Excess events in 28 days per 100,000 (95% CI)		
**Subgroup, exposure**	**Myocarditis**	**Pericarditis**	**Myocarditis and Pericarditis combined**
**Males, 12+, mRNA-1273/mRNA-1273**			
Model 2	4.97 (3.62-6.32)	1.84 (1.04-2.63)	7.74 (6.1-9.37)
Model 3	4.67 (3.38-5.97)	1.89 (1.07-2.7)	7.40 (5.79-9)
**Males, 16-24, BNT162b2**			
Model 2	1.55 (0.7-2.39)	0.08 (-0.35-0.5)	2.38 (1.27-3.49)
Model 3	1.32 (0.4-2.25)	0.01 (-0.47-0.49)	1.89 (0.61-3.18)
**Males, 16-24, BNT162b2/BNT162b2**			
Model 2	5.55 (3.7-7.39)	1.08 (0.26-1.9)	8.30 (6.05-10.54)
Model 3	5.48 (3.44-7.53)	0.85 (-0.11-1.8)	7.85 (5.26-10.44)
**Males, 16-24, BNT162b2/mRNA-1273**			
Model 2	27.49 (14.41-40.56)	4.21 (-0.65-9.07)	37.94 (22.73-53.14)
Model 3	27.93 (14.64-41.21)	…	38.51 (23.07-53.96)
**Males, 16-24, mRNA-1273**			
Model 2	1.75 (-0.2-3.71)	0.86 (-0.51-2.24)	2.55 (0.07-5.03)
Model 3	1.72 (-0.32-3.75)	0.50 (-0.69-1.7)	2.49 (-0.04-5.02)
**Males, 16-24, mRNA-1273/mRNA-1273**			
Model 2	18.39 (9.05-27.72)	7.39 (1.46-13.32)	26.51 (15.38-37.64)
Model 3	18.57 (9.09-28.06)	6.23 (0.11-12.36)	26.47 (15.3-37.63)
**Males, 25-39, BNT162b2**			
Model 2	0.46 (0-0.92)	0.68 (0.25-1.11)	1.15 (0.23-2.08)
Model 3	0.16 (-0.42-0.75)	0.64 (0.18-1.11)	0.87 (-0.2-1.94)
**Males, 25-39, BNT162b2/BNT162b2**			
Model 2	0.59 (0.07-1.1)	1.09 (0.51-1.66)	1.96 (1.1-2.83)
Model 3	0.27 (-0.4-0.95)	1.02 (0.42-1.63)	1.65 (0.7-2.6)
**Males, 25-39, BNT162b2/mRNA-1273**			
Model 2	11.33 (5.59-17.07)	3.03 (0.24-5.83)	19.45 (12.07-26.83)
Model 3	11.18 (5.5-16.87)	3.04 (0.19-5.89)	19.35 (11.97-26.72)
**Males, 25-39, mRNA-1273**			
Model 2	0.16 (-0.55-0.86)	0.71 (-0.09-1.5)	1.17 (-0.08-2.43)
Model 3	-0.09 (-1.1-0.91)	0.62 (-0.21-1.45)	0.73 (-0.75-2.2)

	Excess events in 28 days per 100,000 (95% CI)		
**Subgroup, exposure**	**Myocarditis**	**Pericarditis**	**Myocarditis and Pericarditis combined**
**Males, 25-39, mRNA-1273/mRNA-1273**			
Model 2	8.01 (4.92-11.11)	1.80 (0.4-3.2)	12.11 (8.4-15.83)
Model 3	7.62 (4.63-10.6)	1.65 (0.28-3.03)	11.64 (8-15.28)
**Females, 12+, BNT162b2**			
Model 2	0.15 (0.02-0.28)	0.12 (-0.04-0.27)	0.17 (-0.07-0.42)
Model 3	0.14 (0-0.28)	0.14 (-0.02-0.3)	0.19 (-0.07-0.44)
**Females, 12+, BNT162b2/BNT162b2**			
Model 2	0.09 (-0.09-0.26)	0.20 (0.04-0.36)	0.31 (-0.03-0.66)
Model 3	0.06 (-0.21-0.34)	0.24 (0.07-0.4)	0.25 (-0.26-0.75)
**Females, 12+, BNT162b2/mRNA-1273**			
Model 2	1.44 (0.02-2.87)	2.02 (0.37-3.66)	5.13 (2.49-7.77)
Model 3	1.41 (0.01-2.81)	2.00 (0.35-3.66)	5.04 (2.42-7.66)
**Females, 12+, mRNA-1273**			
Model 2	0.05 (-0.13-0.23)	0.67 (0.24-1.1)	0.92 (0.36-1.48)
Model 3	0.07 (-0.1-0.24)	0.69 (0.25-1.13)	0.93 (0.37-1.5)
**Females, 12+, mRNA-1273/mRNA-1273**			
Model 2	0.48 (0.07-0.89)	1.06 (0.46-1.66)	1.96 (1.1-2.81)
Model 3	0.46 (0.04-0.87)	1.04 (0.43-1.64)	1.88 (1.04-2.72)
**Females, 16-24, BNT162b2**			
Model 2	0.18 (-0.13-0.49)	0.14 (-0.14-0.43)	0.14 (-0.42-0.71)
Model 3	0.13 (-0.26-0.53)	0.21 (-0.09-0.51)	0.12 (-0.51-0.75)
**Females, 16-24, BNT162b2/BNT162b2**			
Model 2	0.57 (-0.01-1.15)	0.12 (-0.14-0.39)	1.05 (0.24-1.85)
Model 3	0.39 (-0.26-1.04)	0.16 (-0.16-0.48)	1.12 (0.25-1.99)
**Females, 16-24, BNT162b2/mRNA-1273**			
Model 2	3.74 (-1.45-8.93)	3.48 (-1.37-8.32)	9.05 (1.10-16.99)
Model 3	3.75 (-1.45-8.96)	3.78 (-1.46-9.01)	9.29 (1.14-17.43)
**Females, 16-24, mRNA-1273**			
Model 2	…	2.05 (-0.27-4.38)	2.41 (0-4.82)
Model 3	…	2.12 (-0.28-4.52)	2.51 (0.02-5.01)

© 2022 Karlstad Ø et al. *JAMA Cardiology.*

	Excess events in 28 days per 100,000 (95% CI)		
**Subgroup, exposure**	**Myocarditis**	**Pericarditis**	**Myocarditis and Pericarditis combined**
**Females, 16-24, mRNA-1273/mRNA-1273**			
Model 2	…	4.94 (0.09-9.78)	7.36 (1.46-13.26)
Model 3	…	5.09 (0.1-10.08)	7.30 (1.44-13.15)
**Females, 25-39, BNT162b2**			
Model 2	0.21 (-0.03-0.45)	0.13 (-0.06-0.31)	0.46 (0.09-0.82)
Model 3	0.18 (-0.07-0.43)	0.13 (-0.07-0.32)	0.47 (0.08-0.86)
**Females, 25-39, BNT162b2/BNT162b2**			
Model 2	0.26 (-0.04-0.55)	0.25 (-0.02-0.53)	0.94 (0.27-1.6)
Model 3	0.22 (-0.22-0.65)	0.25 (-0.03-0.53)	0.91 (0.21-1.61)
**Females, 25-39, BNT162b2/mRNA-1273**			
Model 2	…	2.95 (-0.39-6.28)	4.71 (0.56-8.87)
Model 3	…	2.81 (-0.41-6.03)	4.58 (0.51-8.65)
**Females, 25-39, mRNA-1273**			
Model 2	…	0.48 (-0.2-1.16)	1.41 (0.26-2.56)
Model 3	…	0.45 (-0.2-1.1)	1.43 (0.26-2.6)
**Females, 25-39, mRNA-1273/mRNA-1273**			
Model 2	0.95 (-0.14-2.03)	1.03 (-0.14-2.2)	2.47 (0.73-4.21)
Model 3	0.95 (-0.14-2.04)	1.08 (-0.14-2.29)	2.53 (0.75-4.32)

* Model 2 indicates adjustment for age group, sex, previous SARS-CoV-2 infection, healthcare worker, nursing home resident, comorbidity variables. Model 3 indicates adjustments for Model 2 and calendar time.

Note: For the combined myocarditis and/or pericarditis outcome one of the requirements, hospital stay exceeding 24 hours or inpatient, was ignored.

eTable 6. Myocarditis and Pericarditis Combined Within 28 Days Following a Dose of SARS-CoV-2 Vaccine, Boys 12-15 years

Crude Incidence Rate Ratios (IRR).

Subgroup, exposure	Events	1000 PYR	IR per 1000 PYR	IRR (95%CI)
**Boys 12–15 years**				
Unvaccinated	31	447.9	0.069	1 (ref)
Any[a]	5	15.2	0.330	4.77 (1.85–12.26)
Any[a]/Any[a]	6	6.3	0.959	13.86 (5.78–33.22)

[a] Any indicates either BNT162b2 or mRNA-1273 vaccines.

Abbreviations: PYR follow-up time in person years.

Among boys and girls 12–15 years the 28-day risk period after vaccination yielded very few exposed and few events. A crude incidence rate ratio (IRR) is calculated for any first dose of mRNA vaccine and for any combination of first and second dose of mRNA vaccines.

eTable 7. Myocarditis Within 28 Days of a Positive SARS-CoV-2 Test, According to Sex and Age

Incidence Rate Ratios (IRR) and Excess Events in 28 Days per 100,000 with Positive Test

Subgroup, exposure		Events[a]	1000 PYR	IR per 1000 PYR	IRR (95% CI)	Excess events in 28 days per 100,000 (95% CI)
**Males, 12+**						
No infection		922	9834.5	0.094	1 (ref)	0 (ref)
SARS-CoV-2 infection		42	73.9	0.568	3.96 (1.71-9.17)	3.26 (1.90-4.61)
**Males, 12-15**						
No infection		22	695.1	0.032	1 (ref)	0 (ref)
SARS-CoV-2 infection		0	5.5	…	…	…
**Males, 16-24**						
No infection		234	1321.9	0.177	1 (ref)	0 (ref)
SARS-CoV-2 infection		≤5	14.9		2.99 (1.10-8.12)	1.37 (-0.14-2.87)
**Males, 25-39**						
No infection		267	2439.5	0.109	1 (ref)	0 (ref)
SARS-CoV-2 infection		9	21.4	0.420	5.19 (2.65-10.18)	2.60 (0.85-4.35)
**Males, 40+**						
No infection		399	5378	0.074	1 (ref)	0 (ref)
SARS-CoV-2 infection		29	32	0.906	14.67 (9.94-21.66)	6.47 (4.11-8.84)
**Females, 12+**						
No infection		381	9468.7	0.040	1 (ref)	0 (ref)
SARS-CoV-2 infection		31	70.9	0.437	12.16 (8.37-17.68)	3.08 (1.99-4.17)
**Females, 12-15**						
No infection		≤5	518.1		1 (ref)	0 (ref)
SARS-CoV-2 infection		0	3	…	…	…
**Females, 16-24**						
No infection		48	1200.3	0.040	1 (ref)	0 (ref)
SARS-CoV-2 infection		≤5	14		3.08 (0.42-22.8)	0.37 (-0.44-1.18)
**Females, 25-39**						
No infection		82	2208.1	0.037	1 (ref)	0 (ref)
SARS-CoV-2 infection		9	20.3	0.444	15.34 (7.6-30.96)	3.18 (1.1-5.27)

Subgroup, exposure		Events[a]	1000 PYR	IR per 1000 PYR	IRR (95% CI)	Excess events in 28 days per 100,000 (95% CI)
**Females, 40+**						
No infection		248	5401.9	0.046	1 (ref)	0 (ref)
SARS-CoV-2 infection		20	31.3	0.639	15.37 (9.62-24.55)	4.58 (2.57-6.59)

[a] Data from Norway, with 6 events after positive SARS-CoV-2 test, are not included in the analyses

Abbreviations: PYR follow-up time in person years, IR crude incidence rate, IRR adjusted incidence rate ratio (Model 2: Adjusted for age group, sex, previous SARS-CoV-2 infection, healthcare worker, nursing home resident, comorbidity variables).

eTable 8. Myocarditis Within 7 Days Following a Dose of SARS-CoV-2 Vaccine, According to Sex and Age

Incidence Rate Ratios (IRR) and Excess Events in 7 Days per 100,000 Vaccinees

Subgroup, exposure	Events	1000 PYR	IR per 1000 PYR	IRR (95% CI)	Excess events in 7 days per 100,000 (95% CI)
**Males, 12+**					
Unvaccinated	520	5340.6	0.097	1 (ref)	0 (ref)
AZD1222	≤5	11.4		2.02 (0.28-14.71)	0.09 (-0.15-0.32)
AZD1222/AZD1222	≤5	7.8		2.43 (0.33-17.76)	0.14 (-0.2-0.49)
BNT162b2	16	153.3	0.104	1.18 (0.72-1.95)	0.03 (-0.05-0.12)
BNT162b2/BNT162b2	45	134.5	0.335	4.13 (3.02-5.64)	0.49 (0.34-0.64)
BNT162b2/mRNA-1273	31	6.5	4.754	54.57 (36.29-82.06)	8.95 (5.8-12.1)
mRNA-1273	8	25.1	0.319	3.50 (1.74-7.05)	0.44 (0.11-0.76)
mRNA-1273/mRNA-1273	44	20.3	2.164	25.09 (17.09-36.84)	3.99 (2.81-5.16)
**Males, 16-24**					
Unvaccinated	149	794.6	0.188	1 (ref)	0 (ref)
AZD1222	0	0.2	…	…	…
AZD1222/AZD1222	0	0	…	…	…
BNT162b2	9	17.3	0.519	3.49 (1.77-6.9)	0.71 (0.21-1.21)
BNT162b2/BNT162b2	27	12.3	2.190	12.5 (8.24-18.96)	3.86 (2.4-5.33)
BNT162b2/mRNA-1273	17	1.3	13.028	120.05 (63.45-227.14)	24.77 (13-36.55)
mRNA-1273	≤5	3.2		10.69 (2.82-40.51)	1.64 (-0.23-3.52)
mRNA-1273/mRNA-1273	14	1.9	7.379	38.29 (21.95-66.8)	13.78 (6.56-21)
**Males, 25-39**					
Unvaccinated	146	1440.6	0.101	1 (ref)	0 (ref)
AZD1222	0	0.8	…	…	…
AZD1222/AZD1222	0	0.1	…	…	…
BNT162b2	≤5	29.4		2.79 (0.86-9.03)	0.13 (-0.04-0.29)
BNT162b2/BNT162b2	9	23.5	0.383	3.77 (1.92-7.41)	0.54 (0.16-0.92)
BNT162b2/mRNA-1273	13	2.7	4.767	66.99 (34.88-128.64)	9.01 (4.11-13.9)
mRNA-1273	≤5	8.4		3.77 (0.92-15.4)	0.34 (-0.16-0.83)
mRNA-1273/mRNA-1273	26	6.7	3.898	44.27 (26.86-72.98)	7.31 (4.5-10.12)

Subgroup, exposure	Events	1000 PYR	IR per 1000 PYR	IRR (95% CI)	Excess events in 7 days per 100,000 (95% CI)
**Males, 40+**					
Unvaccinated	206	2657.6	0.078	1 (ref)	0 (ref)
AZD1222	≤5	10.4		2.09 (0.28-15.38)	0.10 (-0.16-0.36)
AZD1222/AZD1222	≤5	7.6		2.24 (0.3-16.46)	0.14 (-0.21-0.49)
BNT162b2	≤5	102.5		0.86 (0.32-2.33)	-0.01 (-0.1-0.08)
BNT162b2/BNT162b2	7	96.8	0.072	1.50 (0.7-3.24)	0.05 (-0.03-0.13)
BNT162b2/mRNA-1273	≤5	2.5		6.95 (0.95-50.97)	0.66 (-0.65-1.98)
mRNA-1273	≤5	12.8		3.67 (1.17-11.5)	0.33 (-0.07-0.72)
mRNA-1273/mRNA-1273	≤5	11.6		5.67 (1.8-17.86)	0.41 (-0.06-0.88)
**Females, 12+**					
Unvaccinated	211	4942.2	0.043	1 (ref)	0 (ref)
AZD1222	≤5	17		2.14 (0.29-15.63)	0.06 (-0.1-0.22)
AZD1222/AZD1222	0	8.5	…	…	…
BNT162b2	11	157.8	0.070	1.67 (0.91-3.08)	0.05 (0-0.11)
BNT162b2/BNT162b2	10	141.1	0.071	2.15 (1.06-4.34)	0.07 (0.01-0.14)
BNT162b2/mRNA-1273	≤5	5.2		28.69 (4.24-194.38)	0.71 (-0.28-1.69)
mRNA-1273	0	24.2	…	…	…
mRNA-1273/mRNA-1273	≤5	20		4.18 (1.33-13.1)	0.22 (-0.04-0.48)
**Females, 16-24**					
Unvaccinated	31	707.1	0.044	1 (ref)	0 (ref)
AZD1222	0	0.6	…	…	…
AZD1222/AZD1222	0	0.1	…	…	…
BNT162b2	≤5	17.1		20.39 (1.85-224.86)	0.11 (-0.1-0.32)
BNT162b2/BNT162b2	≤5	12.8		7.88 (2.31-26.84)	0.39 (-0.06-0.84)
BNT162b2/mRNA-1273	≤5	1.1		210.81 (44.45-999.75)	3.34 (-1.29-7.97)
mRNA-1273	0	2.9	…	…	…
mRNA-1273/mRNA-1273	0	1.9	…	…	…
**Females, 25-39**					
Unvaccinated	42	1269.7	0.033	1 (ref)	0 (ref)
AZD1222	0	2.4	…	…	…
AZD1222/AZD1222	0	0.4	…	…	…
BNT162b2	≤5	28.5		2.37 (0.32-17.78)	0.04 (-0.06-0.13)
BNT162b2/BNT162b2	≤5	23.6		11.05 (2.62-46.66)	0.22 (-0.03-0.47)

Subgroup, exposure	Events	1000 PYR	IR per 1000 PYR	IRR (95% CI)	Excess events in 7 days per 100,000 (95% CI)
BNT162b2/mRNA-1273	0	2.1	…	…	…
mRNA-1273	0	7.6	…	…	…
mRNA-1273/mRNA-1273	≤5	6.1		25.12 (5.78-109.14)	0.6 (-0.23-1.44)
**Females, 40+**					
Unvaccinated	137	2541.6	0.054	1 (ref)	0 (ref)
AZD1222	≤5	14		2.38 (0.32-17.42)	0.08 (-0.11-0.27)
AZD1222/AZD1222	0	8	…	…	…
BNT162b2	9	108.3	0.083	1.65 (0.84-3.25)	0.06 (-0.01-0.14)
BNT162b2/BNT162b2	≤5	103		1.29 (0.47-3.55)	0.02 (-0.04-0.08)
BNT162b2/mRNA-1273	0	2	…	…	…
mRNA-1273	0	12.9	…	…	…
mRNA-1273/mRNA-1273	≤5	11.9		6.24 (0.86-45.60)	0.14 (-0.14-0.41)

Abbreviations: PYR follow-up time in person years, IR crude incidence rate, IRR adjusted incidence rate ratio (Model 2: Adjusted for age group, sex, previous SARS-CoV-2 infection, healthcare worker, nursing home resident, comorbidity variables).

eTable 9. Days From Vaccination to Date of Admission for Vaccinated Myocarditis Cases

Day 0 is Day of Vaccination.

			Events	First quartile	Median	Third quartile
	Age group	Country	N	Day	Day	Day
Myocarditis Both sexes	12+					
		Denmark	64	3	5	7
		Finland	68	5	11	16
		Norway	86	3	5	19
		Sweden	129	3	7	18
Myocarditis Males	12-39					
		Denmark	37	3	4	5
		Finland	34	3	7	15
		Norway	53	3	3	5
		Sweden	54	3	4	9
Pericarditis Both sexes	12+					
		Denmark	62	5	10	18
		Finland	34	10	18	22
		Norway	155	5	12	19
		Sweden	115	8	13	20
Pericarditis Males	12-39					
		Denmark	12	4	6	15
		Finland	5	9	20	20
		Norway	10	2	3	14
		Sweden	19	3	11	15

eTable 10. Distribution of Comorbidities for Total Population at Start of Follow-up and for Myocarditis Cases, by Vaccination Status; Nordic Countries Denmark, Finland, Norway, and Sweden Combined[a]

			Autoimmune disease	Cardiovascular or diabetes	Malignancy	Pulmonary disease	Renal disease	Any comorbidity	Prior covid	Total
**Sex**	**Age**	**Vaccination status**	**%**	**%**	**%**	**%**	**%**	**%**	**%**	**N**
Both	12+	Total population December 27, 2020	3.7	10.3	4.4	3.6	1.2	18.8	2.5	23,122,522
Both	12+	Unvaccinated myocarditis cases	7.1	12.2	5.0	5.5	1.6	24.5	5.3	731
Both	12+	Vaccinated myocarditis cases	4.9	11.8	6.1	5.5	2.3	24.2	5.5	347
Males	12-39	Total population December 27, 2020	1.7	1.1	0.3	2.2	0.3	5.4	2.9	4,609,664
Males	12-39	Unvaccinated myocarditis cases	3.8	3.2	0.0	3.5	0.6	10.2	5.4	314
Males	12-39	Vaccinated myocarditis cases	2.3	2.2	1.1	3.9	1.1	9.0	2.8	178

[a] Definition of comorbidity see Supplemental Table S2

eTable 11. Mortality and Discharge Outcomes Among Myocarditis Cases

Kaplan-Meier estimates combined from all four Nordic countries in meta-analysis with random effects[a].

	Unvacc.[a]	BNT162b2[a]	BNT162b2/ BNT162b2[a]	mRNA-1273[a]	mRNA-1273/ mRNA-1273[a]
**All**	**Percent, (95% CI)**	**Percent, (95% CI)**	**Percent, (95% CI)**	**Percent, (95% CI)**	**Percent, (95% CI)**
Discharged day 4 or later[b]	53.0 (38.0-67.5)	54.0 (32.1-74.6)	53.1 (35.1-70.3)	41.7 (22.5-63.7)	42.6 (31.7-54.3)
Death within 28 days[c]	0.8 (0.3-2.0)	2.3 (0-6.8)	0.2 (0.0-0.4)	NA	4.5 (0.0-13.2)
**Females**					
Discharged day 4 or later[b]	56.9 (43.7-68.9)	48.3 (33.2-63.7)	53.5 (34.7-71.3)	NA	NA
Death within 28 days[c]	2.9 (0.0-18.6)	6.3 (0.0-18.1)	0.2 (0.0-0.7)	4.8 (0.0-13.4)	0 (0-0)
**Males**					
Discharged day 4 or later[b]	50.9 (35.5-66.1)	52.4 (28.9-74.8)	49.8 (31.2-68.5)	37.8 (20.5-58.9)	48.5 (26.5-71.1)
Death within 28 days[c]	0.8 (0.3-1.9)	0 (0-0)	0 (0-0)	NA	4.8 (0.0-13.9)
**Males aged 16-24 years**					
Discharged day 4 or later[b]	45.4 (28.8-63.1)	29.6 (8.4-65.7)	42.1 (20.4-67.4)	NA	NA
Death within 28 days[c]	0 (0-0)	0 (0-0)	0 (0-0)	0 (0-0)	0 (0-0)
**Males aged 25-39 years**					
Discharged day 4 or later[b]	51.8 (29.2-73.7)	44.2 (18.6-73.3)	27.2 (22.1-32.9)	NA	40.6 (12.5-76.5)
Death within 28 days[c]	0 (0-0)	0 (0-0)	0 (0-0)	0 (0-0)	0 (0-0)

[a] Cases included from the 0-28 day time window following vaccination among vaccinated. To combine country-specific estimates the log odds of probabilities were then used as inputs in meta-analysis. Where the confidence interval limits were 0% or 100%, we used the point estimate multiplied by 0.1 or (100-0.1*(100-point estimate)), respectively. If some country-specific estimates were exactly 0, but no country-specific estimate was higher than 20%, then the maximal estimate and upper confidence limit were given as result. Cells marked 'NA' is due to heterogeneity or lack of outcome estimation in individual Nordic countries.

[b] Day of admission is day 0.

[c] Death within 28 days of outcome from any cause.

eFigure 1. Myocarditis in Females Within 28 Days Following SARS-CoV-2 Vaccination

Data from four Nordic countries with pooled estimates. incidence rate ratios with
95% confidence intervals according to age. Model 2 with adjustment for age group, sex, previous SARS-CoV-2 infection, healthcare worker, nursing home resident, comorbidity variables.

Panel: Females 16-24 years.

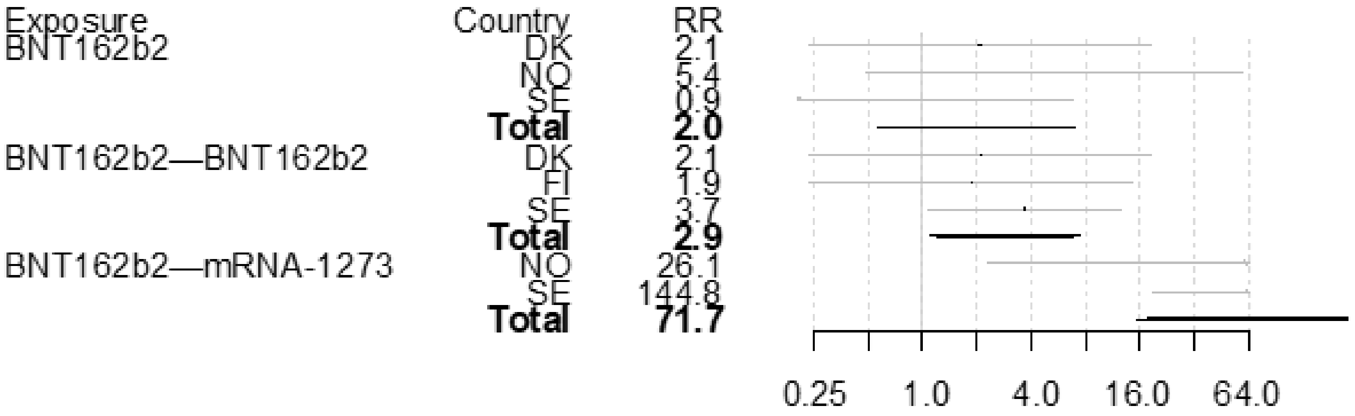

Panel: Females 25-39 years.

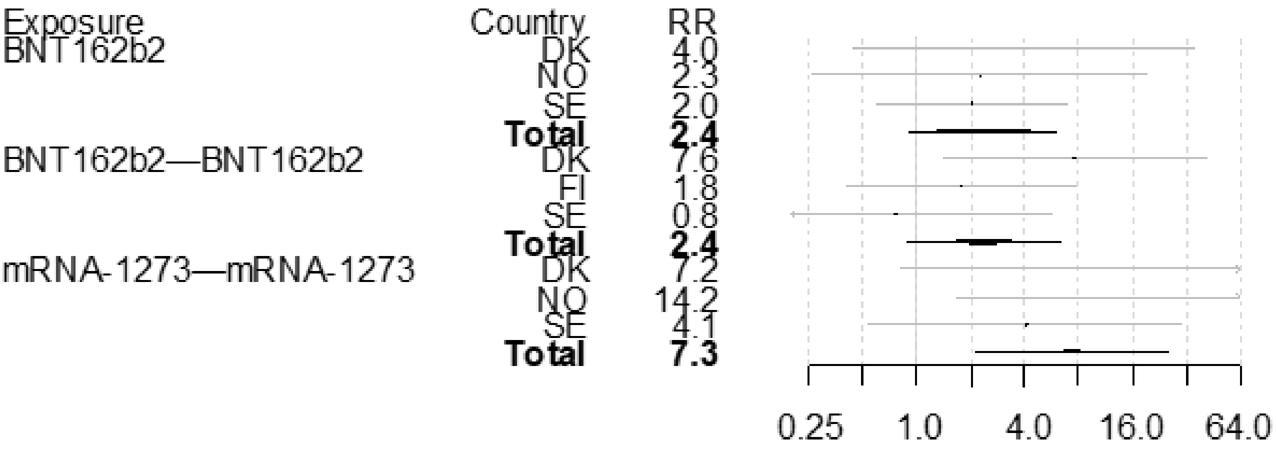

eFigure 2. Meta-analysis Results of Myocarditis Following COVID-19 Vaccination in 4 Nordic Countries

Comparison of Incidence Rate Ratios (IRR) by 3 Models

The models displayed are Model 1: age group and sex, Model 2: Model 1 and previous SARS-CoV-2 infection, healthcare worker, nursing home resident, comorbidity variables, and Model 3: Model 2 and calendar time).

eFigure 2: Panel: Males 12+ years.

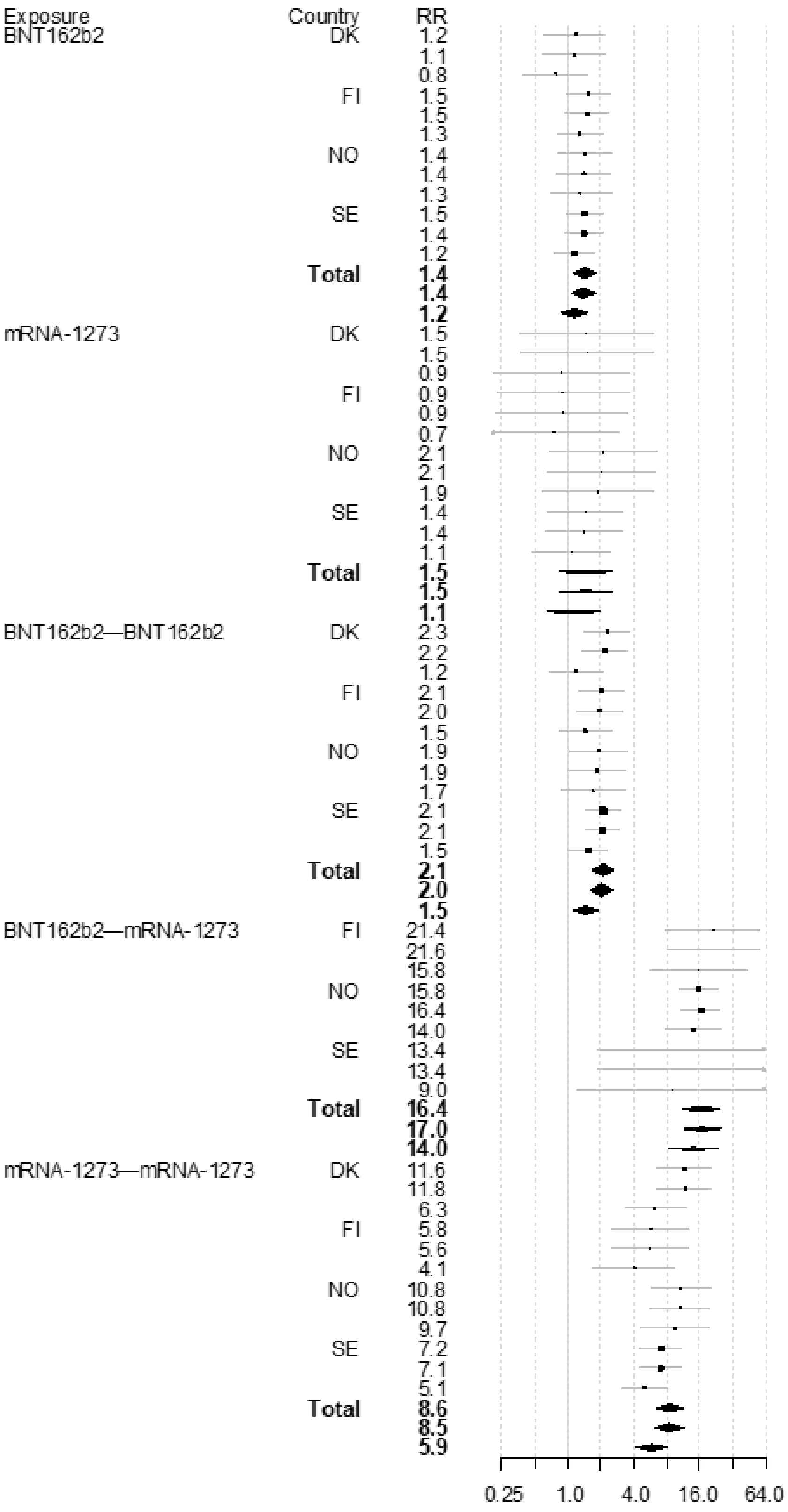

eFigure 2 Panel: Males 16 to 24 years.

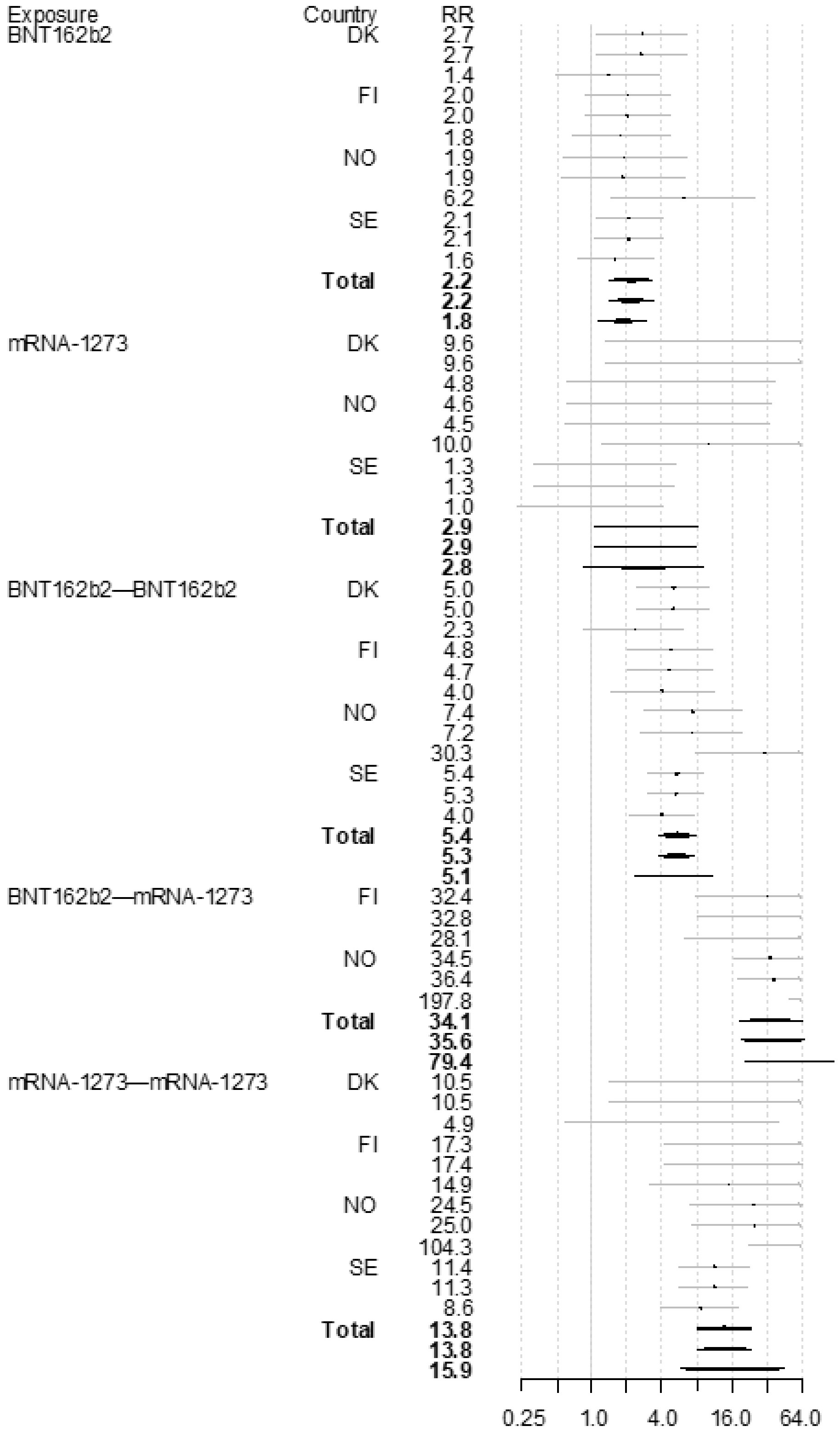

eFigure 2 Panel: Males 25 to 39 years.

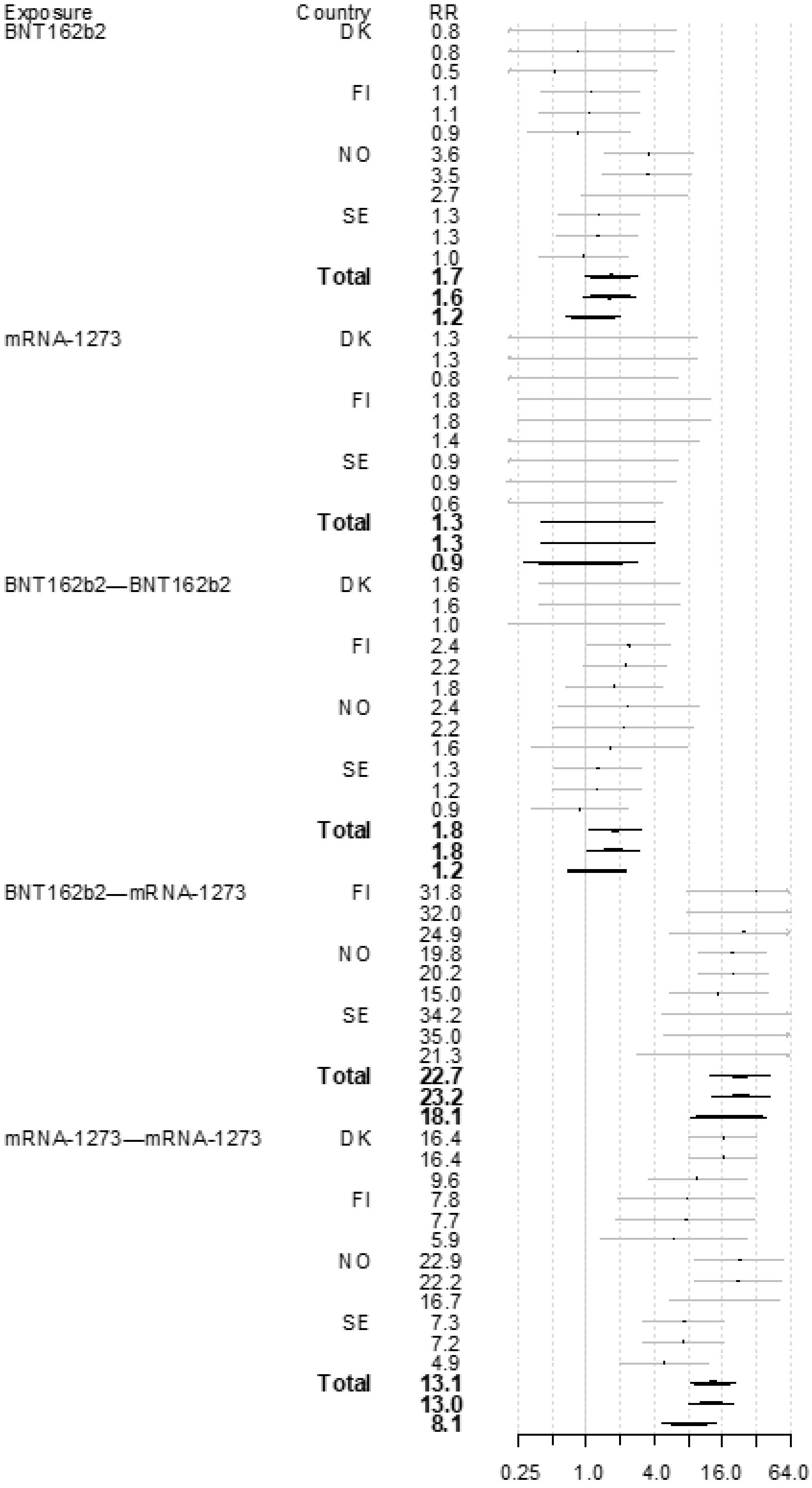

eFigure 2 Panel: Females 12+ years.

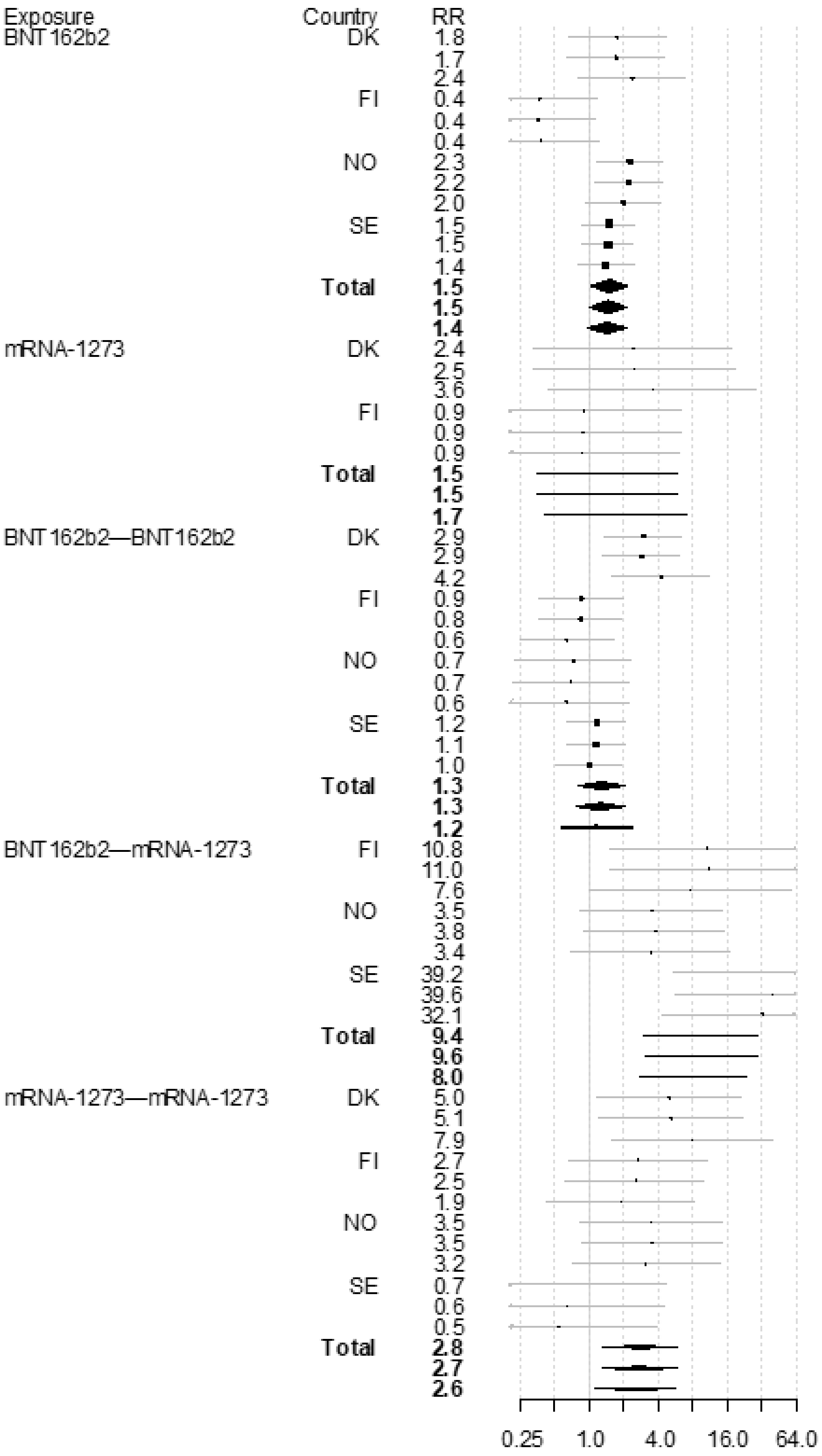

eFigure 2 Panel: Females 16 to 24 years.

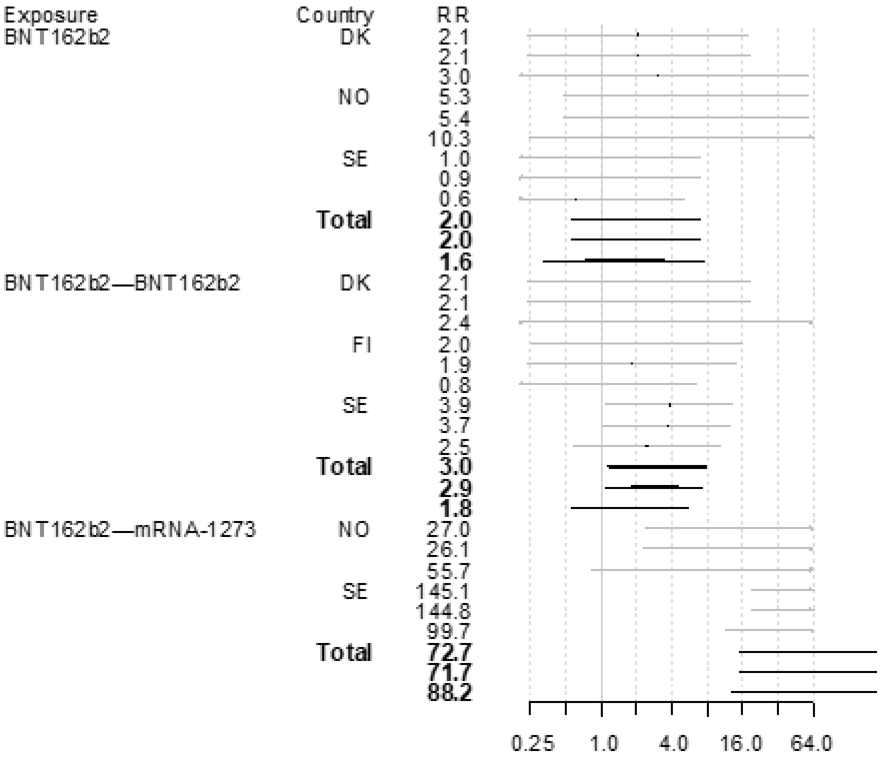

eFigure 2 Panel: Females 25 to 39 years.

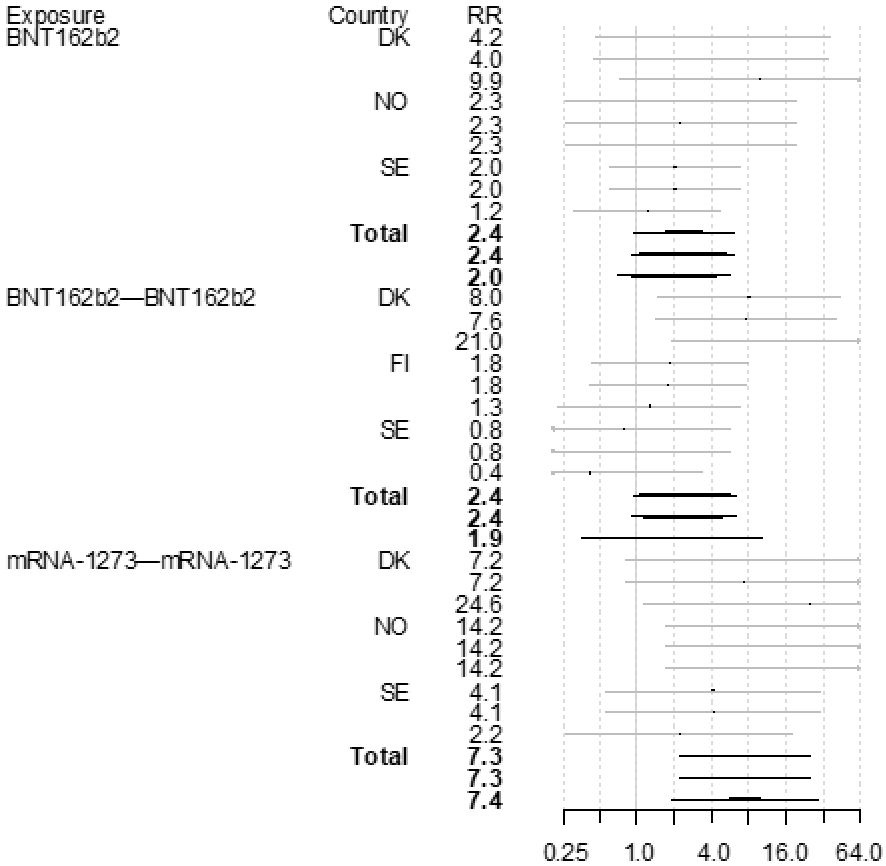

eReferences

1. Schmidt M, Pedersen L, Sørensen HT. The Danish Civil Registration System as a tool in epidemiology. *Eur J Epidemiol*. 2014;29(8):541-549. doi:10.1007/S10654-014-9930-3

2. Krause TG, Jakobsen S, Haarh M, Mølbak K. The Danish vaccination register. *Eurosurveillance*. 2012;17(17):20155. doi:10.2807/ESE.17.17.20155-EN

3. Schmidt M, Schmidt SAJ, Sandegaard JL, Ehrenstein V, Pedersen L, Sørensen HT. The Danish National Patient Registry: a review of content, data quality, and research potential. *Clin Epidemiol*. 2015;7:449-490. doi:10.2147/CLEP.S91125

4. Voldstedlund M, Haarh M, Mølbak K, the MiBa Board of Representatives C. The Danish Microbiology Database (MiBa) 2010 to 2013. *Eurosurveillance*. 2014;19(1):20667. doi:10.2807/1560-7917.ES2014.19.1.20667

5. Baum U, Sundman J, Jääskeläinen S, Nohynek H, Puumalainen T, Jokinen J. Establishing and maintaining the National Vaccination Register in Finland. *Euro Surveill*. 2017;22(17). doi:10.2807/1560-7917.ES.2017.22.17.30520

6. Finnish National Infectious Diseases Register - Infectious diseases and vaccinations - THL. Accessed November 5, 2021. https://thl.fi/en/web/infectious-diseases-and-vaccinations/surveillance-and-registers/finnish-national-infectious-diseases-register

7. Care Register for Health Care - THL. Accessed November 5, 2021. https://thl.fi/en/web/thlfi-en/statistics-and-data/data-and-services/register-descriptions/care-register-for-health-care

8. Population Information System | Digital and population data services agency. Accessed November 5, 2021. https://dvv.fi/en/population-information-system

9. Register of Social assistance - THL. Accessed November 5, 2021. https://thl.fi/en/web/thlfi-en/statistics-and-data/data-and-services/register-descriptions/social-assistance

10. Quality description - kela.fi. Accessed November 5, 2021. https://www.kela.fi/web/en/quality-description18?inheritRedirect=true#year--106

11. Terhikki Register - valvira englanti - Valvira. Accessed November 5, 2021. https://www.valvira.fi/web/en/healthcare/professional_practice_rights/terhikki_register

12. Emergency preparedness register for COVID-19 (Beredt C19) - Norwegian Institute of Public Health. Accessed November 5, 2021. https://www.fhi.no/en/id/infectious-diseases/coronavirus/emergency-preparedness-register-for-covid-19/

13. Trogstad L, Ung G, Hagerup-Jenssen M, Cappelen I, Haugen IL, Feiring B. The norwegian immunisation register - SYSVAK. *Eurosurveillance*. 2012;17(16). doi:10.2807/ESE.17.16.20147-EN

14. Bakken IJ, Ariansen AMS, Knudsen GP, Johansen KI, Vollset SE. The Norwegian Patient Registry and the Norwegian Registry for Primary Health Care: Research potential of two nationwide health-care registries: *https://doi.org/101177/1403494819859737*. 2019;48(1):49-55. doi:10.1177/1403494819859737

15. State Register of Employers and Employees (Aa-registeret) - nav.no. Accessed November 5, 2021. https://www.nav.no/en/home/employers/nav-state-register-of-employers-and-employees

16. Iplos-registeret - Helsedirektoratet. Accessed November 8, 2021. https://www.helsedirektoratet.no/tema/statistikk-registre-og-rapporter/helsedata-og-helseregistre/iplos-registeret

17. Chrapkowska C, Galanis I, Kark M, et al. Validation of the new Swedish vaccination register – Accuracy and completeness of register data. *Vaccine*. 2020;38(25):4104-4110. doi:10.1016/J.VACCINE.2020.04.020

18. Rolfhamre P, Janson A, Arneborn M, Ekdahl K. SmiNet-2: Description of an internet-based surveillance system for communicable diseases in Sweden. *Eurosurveillance*. 2006;11(5):15-16. doi:10.2807/ESM.11.05.00626-EN

19. Ludvigsson JF, Andersson E, Ekbom A, et al. External review and validation of the Swedish national inpatient register. *BMC Public Heal 2011 111*. 2011;11(1):1-16. doi:10.1186/1471-2458-11-450

20. The National Patient Register - Socialstyrelsen. Accessed November 5, 2021. https://www.socialstyrelsen.se/en/statistics-and-data/registers/register-information/the-national-patient-register/

21. Barlow L, Westergren K, Holmberg L, Talbäck M. The completeness of the Swedish Cancer Register: a sample survey for year 1998. *Acta Oncol*. 2009;48(1):27-33. doi:10.1080/02841860802247664

22. The Swedish Cancer Register - Socialstyrelsen. Accessed November 5, 2021. https://www.socialstyrelsen.se/en/statistics-and-data/registers/register-information/swedish-cancer-register/

23. Wettermark B, Hammar N, Fored C, et al. The new Swedish Prescribed Drug Register--opportunities for pharmacoepidemiological research and experience from the first six months. *Pharmacoepidemiol Drug Saf*. 2007;16(7):726-735. doi:10.1002/PDS.1294

24. The Swedish Prescribed Drug Register - Socialstyrelsen. Accessed November 5, 2021. https://www.socialstyrelsen.se/en/statistics-and-data/registers/register-information/the-swedish-prescribed-drug-register/

25. Brooke HL, Talbäck M, Hörnblad J, et al. The Swedish cause of death register. *Eur J Epidemiol 2017 329*. 2017;32(9):765-773. doi:10.1007/S10654-017-0316-1

26. Ludvigsson J, Almqvist C, Bonamy A, et al. Registers of the Swedish total population and their use in medical research. *Eur J Epidemiol*. 2016;31(2):125-136. doi:10.1007/S10654-016-0117-Y

27. Registret över insatser till äldre och personer med funktionsnedsättning - Socialstyrelsen. Accessed November 5, 2021. https://www.socialstyrelsen.se/statistik-och-data/register/alla-register/aldre-och-personer-med-funktionsnedsattning/

28. Ludvigsson JF, Svedberg P, Olén O, Bruze G, Neovius M. The longitudinal integrated database for health insurance and labour market studies (LISA) and its use in medical research. *Eur J Epidemiol 2019 344*. 2019;34(4):423-437. doi:10.1007/S10654-019-00511-8

29. FINLEX ® - Translations of Finnish acts and decrees: 1227/2016 English.
